# Players’, Head Coaches', And Medical Personnels' Knowledge, Understandings and Perceptions of Injuries and Injury Prevention in Elite-Level Women’s Football in Ireland

**DOI:** 10.1186/s40798-023-00603-6

**Published:** 2023-07-29

**Authors:** Dan Horan, Seamus Kelly, Martin Hägglund, Catherine Blake, Mark Roe, Eamonn Delahunt

**Affiliations:** 1 School of Public Health, Physiotherapy and Sports Science, University College Dublin, Dublin, Ireland; 2 Football Research Group, Linköping University, Linköping, Sweden; 3 Division of Physiotherapy, Department of Health, Medicine and Caring Sciences, Linköping University, Linköping, Sweden; 4 Institute for Sport and Health, University College Dublin, Dublin, Ireland; 5 Department of Sport, Leisure & Childhood Studies, Munster Technological University, Cork, Ireland

**Keywords:** Football, Sports medicine, Injuries, Women, Prevention

## Abstract

**Background:**

To manage injuries effectively, players, head coaches, and medical personnel need to have excellent knowledge, attitudes, and behaviours in relation to the identification of risk factors for injuries, the implementation of injury prevention initiatives, as well as the implementation of effective injury management strategies. Understanding the injury context, whereby specific personal, environmental, and societal factors can influence the implementation of injury prevention initiatives and injury management strategies is critical to player welfare. To date, no qualitative research investigating the context of injuries, has been undertaken in elite-level women’s football. The aim of our study was to explore the knowledge, attitudes, and behaviours of players, head coaches, and medical personnel in the Irish Women’s National League (WNL) to injury prevention and injury management.

**Methods:**

We used qualitative research methods to explore the knowledge, attitudes, and behaviours of players, head coaches, and medical personnel in the Irish WNL to injury prevention and injury management. Semi-structured interviews were undertaken with 17 players, 8 medical personnel, and 7 head coaches in the Irish WNL. The data were analysed using thematic analysis. Our study is located within an interpretivist, constructivist research paradigm.

**Results:**

The participants had incomplete knowledge of common injuries in elite-level football, and many held beliefs about risk factors for injuries, such as menstrual cycle stage, which lacked evidence to support them. Jumping and landing exercises were commonly used to reduce the risk of injuries but evidence-based injury prevention exercises and programmes such as the Nordic hamstring curl, Copenhagen adduction exercise, and the FIFA 11+ were rarely mentioned. Overall, there was dissatisfaction amongst players with their medical care and strength and conditioning (S & C) support, with resultant inadequate communication between players, head coaches, and medical personnel.

**Conclusion:**

Poor quality and availability of medical care and S & C support were considered to be a major obstacle in the effective implementation of injury risk reduction strategies and successful return-to-sport practices. More original research is required in elite-level women’s football to explore injury risk factors, injury prevention initiatives, and contextual return-to-sport strategies, so that players, head coaches, and medical personnel can use evidence that is both up-to-date and specific to their environment.

**Supplementary Information:**

The online version contains supplementary material available at 10.1186/s40798-023-00603-6.

## Key points

- This is the first study to investigate the context of injuries in elite-level women’s football.
- Elite-level women’s football players have a poor understanding of the most common injuries sustained during training and match-play.
- Players, head coaches, and medical personnel have beliefs about injury risk factors that are not evidence-based.
- Unqualified and inexperienced medical and S & C personnel, who do not have the skillset to create a collaborative, high-performance environment in unison with the coaching staff, are commonly used by clubs in the Irish Women’s National League.
- More original research is required in elite-level women’s football to explore risk factors for injury, injury prevention initiatives and contextual return-to-sport strategies so that players, head coaches, and medical personnel can use evidence that is both up-to-date and specific to their environment.

## Background

Women’s football is gaining in popularity all the time with increasing participation rates, professionalism, viewership numbers, and financial investment from governing bodies [1, 2]. UEFA has reported that there has been a 50% increase in the number of professional women football players since 2017 [2]. Moreover, over the same time period, there has been a 10% increase in the total financial investment in women’s football by national associations across Europe, as well as the introduction of the first fully professional women’s league in England [2].

The physical demands of elite-level women’s football have also increased [3, 4]. Analyses of the physical demands of the 2019 Women’s World Cup highlighted that on average, distances covered by teams in the highest speed zone increased by 30% when compared to the 2015 World Cup [4]. Frequent exposure to more physically demanding training and match play increases the susceptibility of athletes to injuries [5]. The injury burden incurred by elite-level women football players and clubs can be substantial, ranging from 127/1000 to 216/1000 h—as reported in published studies [6–8]. The mean days lost per injury in these studies ranged from 23 to 35 days, highlighting the moderate to severe nature of many of the injuries incurred by elite-level women football players. Consequently, to manage injuries effectively from initial diagnosis through to return to full match play, players, coaches, and medical personnel need to have excellent knowledge, attitudes, and behaviours in relation to the identification of risk factors for injury, the implementation of injury prevention initiatives, and the implementation of effective injury management strategies.

The quality and size of the medical, sports science, and strength and conditioning (S & C) teams supporting elite-level women’s football clubs vary considerably between leagues and countries [6, 7, 9]. The clarity of communication between the different stakeholders and the leadership style of the head coach could have an influence on injury incidence rates (IIRs) and injury burden at the elite level of the women’s game [10, 11]. Finch [12], Bolling et al. [13], and Verhagen et al. [14] have all emphasised the importance of understanding the injury context, whereby specific personal, environmental, and societal factors can influence the implementation of injury prevention initiatives and the implementation of effective injury management strategies.

To gain a clearer understanding of the injury context in women’s football and to assist in the design of more user-friendly and effective injury surveillance systems and injury prevention initiatives [15, 16], the perceptions and understandings of players, coaches, and medical personnel need to be explored [17–20]. Qualitative research enables the analysis of the understandings, beliefs, and behaviours of participants [21] and has been used effectively in sports medicine to provide a better understanding of the environment from the perspective of athletes, coaches, and medical personnel [18, 22]. To date, no qualitative research investigating the context of injury, including injury prevention and injury management practices has been undertaken in elite-level women’s football. The aim of our study was to use qualitative research methods to explore the knowledge, attitudes, and behaviours of players, head coaches, and medical personnel in the Irish Women’s National League (WNL) to injury prevention and injury management.

## Methods

### Study Design

Our study is located within an interpretivist, constructivist research paradigm where knowledge is viewed as a co-constructed activity involving the researcher, the researched (i.e., the research participants), and the research team [23, 24]. We adhered to the recommendations detailed in the Consolidated Criteria for Reporting Qualitative Research [25] (Additional file 1).

### Participants

In line with recommendations [21, 22, 26], we aimed to recruit a sample of participants with diverse perspectives, backgrounds, and experiences. Potential participants deemed appropriate were contacted by the primary researcher (DH). They all received an introductory telephone call and an email from the primary researcher that provided an initial overview of the study and an invitation to participate. They were also provided with opportunity to contact the primary researcher at a future date prior to participation if they required any further information about the study. The inclusion criteria were that the participants, at the time of the interview, had to be formally affiliated with a team in the WNL in Ireland. Specifically, all the medical personnel had to be currently, or in the previous season, engaged in the provision of medical care to a team in the WNL in Ireland. Head coaches had to be currently affiliated with a team in the WNL in Ireland, whilst players had to be registered with a team in the WNL in Ireland. Representatives from one of the clubs did not participate in the research due to their unavailability. Amongst those satisfying criterion-based purposive sampling criteria, head coaches (*n* = 7), elite female players (17), and medical personnel (*n* = 8) representing clubs in the WNL in Ireland agreed to participate (Table 1). The WNL is the highest playing division for women football players in Ireland; there were 9 clubs in the league at the time of this study, with 25 players per squad. All the participants provided verbal informed consent and received information concerning the ethical considerations, issues of confidentiality, and assurances of anonymity.

**Table 1:** Demographic characteristics of participants

Participants	N	Mean age, years	Gender (M/F)
Players	17	26.9	17F
Head coaches	7	44.7	7 M
Medical personnel	8	29.1	6 M; 2F

### Data Collection

We developed a semi-structured interview guide (Additional file 2) through a process of theoretical and pragmatic problematisation [27] and by consulting recommendations in the literature [21, 22]. Whilst a semi-structured interview guide can provide structure to interviews, the first author adopted a conversational and flexible approach [28, 29], involving clarification and elaboration probes; this facilitated an in-depth exploration of the research aims [30]. This flexibility allowed the participants to speak freely, share additional insights, digress appropriately from the interview guide, and consequently enhanced the fluency of the interview, thereby enabling the acquirement of rich data and ensuring a systematic process of consistent data acquisition across all interviews [31]. During data collection, our research team held regular debriefing sessions that provided opportunities for us to discuss and reflect upon the interview process.

The first author conducted the interviews between March 2020 and June 2020. Due to COVID-19, 28 of the interviews were video-based and took place online. The average length of the interviews was 47 min (range 28–111 min). Data collection ceased when no new themes were identified indicating that data and meaning saturation was attained [28, 32].

### Data analysis

The interview data were analysed using Braun & Clarke’s approach to Reflexive Thematic Analysis [28, 29]. The first author adopted the qualitative stance of ‘familiarisation’ post-transcription, which involved listening to the audio recorded files and multiple readings of the verbatim transcripts to accurately comprehend the data corpus. The data were inductively analysed using open codes with an emphasis on deriving semantic and then latent codes. Subsequently, initial themes were developed from the coded data and then reviewed. Themes were further refined, defined, named, and then analysed for contradictory perspectives, multiple meanings, and novel insights before being structured into a framework of higher-order themes. We conceptualised themes as patterns of shared meaning united by a central concept, developing out of the analytic process following coding [28]. In producing the paper, continuous drafting and redrafting were an integral part of the data analysis process and the refinement of the interpretations that are presented [28, 29].

## Results

Table 2 outlines the themes and sub-themes that were developed from the data. Additional file 3 includes example quotes supporting the theme “Injuries” and its associated sub-themes (common injuries; player availability; risk factors). Additional file 4 includes example quotes supporting the theme “Prevention of Injuries” and its associated sub-themes (monitoring; injury surveillance; injury prevention strategies). Additional file 5 includes example quotes supporting the theme “Injury Management” and its associated sub-themes (qualifications of medical personnel; availability of medical personnel; knowledge and competencies of medical personnel; communication amongst players, head coaches, and medical personnel). All quotes are categorised according to the three domains of: (1) knowledge; (2) attitudes; (3) practices. Additional file 6 includes NVIVO coding for all themes and sub-themes.

**Table 2:** Themes and sub-themes

Main theme	Injuries			
Sub-themes	Common injuries	Player availability	Risk factors	

Main theme	Prevention of injuries			
Sub-themes	Monitoring	Injury surveillance	Injury prevention strategies	

Main theme	Injury management			
Sub-themes	Qualifications of medical personnel	Availability of medical personnel	Knowledge and competencies of medical personnel	Communication amongst players, coaches, and medical personnel

### Injuries

Nineteen of the thirty-two participants mentioned common injuries that are incurred by players in the WNL (Additional file 6: Table S1). Almost half of the participants (3 coaches, 3 medical personnel, 9 players) believed that anterior cruciate ligament (ACL) tears were one of the most common injuries. Nine of the participants (1 coach, 2 medical personnel, 6 players) believed that lateral ankle sprains were a common injury, whilst nine (2 coaches, 3 medical personnel, and 4 players) also believed that hamstring muscle injuries were a commonly incurred injury. A smaller number of participants believed that quadriceps tears, adductor tears, concussions, and knee medial collateral ligament sprains were commonly incurred injuries. Only two of the participants (2 medical personnel) mentioned knee meniscus and cartilage injuries.

Half of the participants mentioned the negative effect that injuries can have on players’ availability for matches, whilst a fifth of them mentioned the effect of injuries on player availability for training and its influence on match preparation (Additional file 6: Table S2). Half of the participants (2 coaches, 4 medical personnel, 10 players) believed that the menstrual cycle and its associated hormonal changes might be a specific risk factor for injuries (Additional file 6: Table S3). The second most cited risk factor for injury was the conflict that arises with college football scheduling at certain times of the season. Other frequently mentioned risk factors for injury were, the poor athletic development of the players, the fact that players in the WNL often participate in multiple sports, and errors in training load management. Only four of the participants (1 coach, 2 medical personnel, 1 player) mentioned previous injury as a risk factor for a recurrent or subsequent injury.

### Prevention of injuries

Over one third of the participants (1 coach, 4 medical personnel and 7 players) mentioned that training load monitoring was important for the prevention of injuries, whilst a quarter of them (1 coach, 3 medical personnel, 4 players) believed that wellness monitoring, including monitoring mood state, energy levels, and nutrition status, had a role to play in the prevention of injuries (Additional file 6: Table S4). Only two of the participants (2 medical personnel) mentioned using regular musculoskeletal screening tests such as the sit and reach and adductor squeeze test to assist in injury risk screening (Additional file 6: Table S4).

One third of the participants (1 coach, 2 medical personnel, 8 players) considered the use of an injury surveillance system to play an important role in the development and implementation of injury prevention initiatives (Additional file 6: Table S5). Three of the participants believed that ongoing injury surveillance enabled the identification of injury risk factors, as well as the most common locations and types of injuries and recurrent injuries. Three of them (1 medical personnel and 2 players) also believed that accurate injury surveillance data could be used as evidence in the request for improved funding towards the provision of medical care from sport’s governing bodies. However, one of the medical personnel articulated that clubs may not appreciate the value in spending time and scarce resources on injury surveillance when there are more perceived pressing needs (e.g., marketing and communications) (Additional file 6: Table S5).

Seventeen of the participants (5 coaches, 2 medical personnel, 10 players) believed that pre-activation exercises using mini-bands were important for the prevention of injuries (Additional file 6: Table S6). Thirteen of the participants thought that modifying training sessions for different players based on their injury history or fixture congestion was an important injury risk mitigation strategy. Nine of the participants (2 coaches, 3 medical personnel, 4 players) highlighted that jumping and landing exercises could be used to reduce the risk of knee injuries. Six of them (2 medical personnel, 4 players) believed that regular exposure to high-speed running and sprinting was critical to prevent new injuries and reduce the risk of re-injuries (Additional file 6: Table S6). Five of the participants reported that proprioception exercises could be used to prevent ankle injuries, whilst three of them (1 coach, 2 medical personnel) thought that regular massage was an effective injury prevention strategy despite acknowledging the lack of evidence to support this view. Two coaches and one medical personnel described using modified versions of the FIFA 11+ programme. Only one of the participants (1 medical personnel) mentioned using Nordic hamstring curls, isometric exercises, or the Copenhagen adduction exercise to prevent injuries (Additional file 6: Table S6).

### Injury Management

Eight of the participants (2 coaches, 1 medical personnel, 5 players) believed that financial constraints at WNL clubs, leading to the use of student medical personnel in place of qualified practitioners, was putting players’ health at risk (Additional file 6; : Table S7). Eleven of the participants (1 coach, 4 medical personnel, 6 players) thought that the lack of medical staff availability at all weekly training sessions had a negative impact on the management of injuries, with four of them highlighting the importance of medical staff being available to assess injuries on the day of their occurrence. Five of the participants (1 medical personnel, 4 players) reported that some clubs shared medical personnel at matches leading to frustration amongst players and lack of confidence in the decision-making of the medical personnel (Additional file 6: Table S8).

A third of the participants (2 medical personnel, 9 players) highlighted the importance of the knowledge of medical personnel regarding return-to-play strategies (Additional file 6: Table S9). Nine of them (1 coach, 3 medical personnel, 5 players) believed that the interpersonal and communication skills of medical personnel were key attributes in the management of injuries. Four of the participants (1 coach, 1 medical personnel, 2 players) thought that the medical personnel had a duty of care to make decisions that are in the best interests of players, although some players expressed a feeling that this was not always the case. Two of the participants (1 medical personnel, 1 player) mentioned that concussion management was an important area of competence for medical personnel despite suggesting that some medical personnel were not competent in this area. Only two players mentioned the importance of injury prevention knowledge for medical personnel (Additional file 6: Table S9).

Nine of the participants (5 medical personnel, 4 coaches) mentioned the importance of regular clear communication between the coach and the medical personnel in injury management (Additional file 6: Table S10). Coaches described weighing up the information they received from medical personnel and players, as well as their own judgement on injury risk and game importance when considering how to manage injured players. Coaches and medical personnel also emphasised the importance of trust from players that communicating honestly with medical personnel would not negatively affect their availability for match selection. One coach described how a player’s importance to team performance and potential success influenced the pressure he exerted on the medical personnel to convince the player she was fit to play when injured. Some of the players thought that the more experienced players were trusted to make their own decisions regarding injury and availability to play, whereas for less experienced players the medical personnel and coach would make the decision (Additional file 6: Table S10).

## Discussion

This study explored the knowledge, attitudes, and behaviours of players, head coaches, and medical personnel in the Irish WNL in relation to injury prevention and injury management. The main themes and sub-themes provide insights into the knowledge of players, head coaches, and medical personnel of the most common injuries incurred by elite-level women football players, as well as insights into their attitudes and behaviours towards the prevention and management of injuries. The sub-themes also emphasise the importance of the qualifications of medical personnel, the availability of medical personnel, the knowledge and competencies of medical personnel, as well as the clarity and quality of communication between the player, coach, and medical personnel (Table 2).

### Knowledge, Attitudes, and Behaviours Towards Injury

Some of the players, coaches, and medical personnel displayed knowledge regarding the most commonly incurred injuries, however a majority of participants were uncertain about which injuries were most frequently incurred. In a two-season prospective injury surveillance study, Horan et al. [6] reported that lateral ankle sprains and hamstring injuries were the two most frequently incurred injuries in the WNL.

Almost half (47%) of the participants believed that ACL tears were one of the most common injuries incurred by players in the WNL, even though they did not rank in the top six most common injury types incurred during the 2018 and 2019 seasons [6]. Albeit, ACL tears were the injury type associated with the highest injury burden, accounting for 28% of all the time lost due to injury [6]. Walden et al. [33] in their 3-cohort study of ACL injuries in elite-level men’s and women’s Swedish football and European men’s first leagues from 11 national associations, reported that the incidence rate of ACL injuries was higher in women’s football; however they noted that ACL injuries were an uncommon injury in women’s and men’s football when compared to thigh muscle injuries and ankle sprains. Only two of the participants (2 medical personnel) mentioned knee meniscus and cartilage injuries although they had the third highest injury incidence rate and the second highest injury burden during the 2018 and 2019 WNL seasons [6].

Many of the participants highlighted the negative impact that injuries have on players’ availability for matches. Despite some evidence in international-level women’s football [34], there is no published research documenting the relationship between player availability and team success in elite-level women’s club football. Eirale et al. [35] and Hägglund et al. [36] have shown that player availability for matches affects team success in elite-level men’s club football, so it is likely that team success in elite-level women’s club football is similarly affected by player availability. Despite limited evidence to support their view [37, 38], half of the study participants, including the majority of players, believed the hormonal changes associated with the menstrual cycle were a risk factor for injury. There is some preliminary evidence suggesting that the incidence rate and type of injuries may vary across the eumenorrheic menstrual cycle [37], and that symptoms associated with the menstrual cycle affect athletes’ training and competition availability [39], but robust studies confirming the relationship between menstrual cycle phase and injury occurrence do not currently exist.

One of the perceived risk factors for injury most commonly mentioned by the participants was fixture congestion as a result of players participating in college football alongside the WNL. Although evidence for fixture congestion as a risk factor for injury in elite-level men’s football exists [40–42], there is currently no published evidence to support this association in women’s football; although we suggest it is likely that fixture congestion is also a risk factor in women’s football particularly when considering the increases in the match running demands in recent years [3, 4]. The potential link between deficits in the athletic development of female footballers and injury was also mentioned by several of the medical personnel and players but, interestingly, not by the head coaches suggesting that either they did not consider S & C input to be effective in helping to reduce injury risk or they were unfamiliar with its application in women’s football. The lack of S & C support and expertise available at club level amongst international level women’s football players was highlighted in a recent survey of players participating in the World Cup [9]. Additionally, Parsons et al. [43] have suggested that gender inequities in the provision of support services could be a risk factor for injury. However, despite some evidence in men’s football and adolescent female football that strength training reduces injury incidence rates[44], research needs to be undertaken in elite-level senior women’s football to confirm the relationship between strength training and injury risk reduction [45].

Perceived errors in training load management (i.e., either under training or overtraining) was a source of frustration amongst several of the players and was considered to be a risk factor for injury. Surprisingly, more than half of the head coaches and medical personnel did not discuss training load and possible injury risk, suggesting a disconnect between the different stakeholders’ perceptions of the role of training load management in injury risk mitigation. Gabbett et al. [46], Gabbett [47], and Drew and Finch [48] have suggested that overloading and underloading athletes are both risk factors for injury, although Kalkhoven et al. [49] highlight that understandings of the causal pathways between training loads and sports injury are still in their infancy. A systematic review by Costa et al. [50] on training load monitoring in college-level and adult elite-level women’s football showed that only one study [51] reported a relationship between training load and injuries—consequently the relationship between training load and injury risk in women’s football is unclear. Impellizzeri et al. [52] suggest that practitioners should use their experience and rely on the traditional principles of gradual overload progression and modify training loads based on how athletes are responding.

Harkness–Armstrong et al. [53] discussed the current lack of evidence regarding the peak physical characteristics or true worst-case scenarios of match-play in women’s football. This makes it difficult for coaches to accurately design training sessions that prepare players adequately for all the demands of match-play. They also suggest future research should analyse the technical, tactical and physical demands of the women’s game in combination rather than in isolation from each other in order to more accurately capture the full demands of the game. Despite a lack of consensus on thresholds for high-speed running and sprinting speeds at different levels of women’s football, Vescovi et al. [3] have highlighted the increasing demands in high-speed running and sprinting between youth-level, college-level and senior-level women’s football. To mitigate injury risk, coaches, fitness, and medical personnel in elite-level women’s football need to be cognisant of this when players are transitioning from youth-level and college-level football to the higher running demands of senior-level football and should implement comprehensive S & C programmes and periodised training plans. Consequently, this requires governing bodies and clubs to invest in appropriately qualified staff so that UEFA’s aspiration to provide higher professional standards and protection for players comes to fruition [54].

Despite some evidence in women’s football that previous injury is a risk factor for injury [55, 56], only four of the participants, including one head coach and two of the medical personnel, mentioned this. This suggests that head coaches in the WNL may not focus on players’ injury histories when recruiting players, which could potentially impact injury incidence rates from season-to-season. One of the other risk factors for injury in women’s football, hamstring – quadriceps ratio was only mentioned by one of the participants, whilst other evidence-based risk factors such as higher BMI, increased joint laxity, trait anxiety, negative life event stress and daily hassle were not mentioned by any of the participants [57–62]. This may be because players, head coaches, and medical personnel in women’s football intuitively recognise the complexity of injury risk in practice and understand that focusing on a pattern of interactions such as such as training load, neuromuscular capacity, level of anxiety, and muscle strength is more effective than focusing on individual risk factors in isolation [61, 62], although this view was not articulated by any of the participants.

### Knowledge, Attitudes, and Behaviours Towards Prevention of Injury

Training load monitoring and wellness monitoring (e.g., mood state, energy levels, nutrition status) were the most frequently mentioned injury prevention strategies by the participants, although only one of the seven head coaches mentioned each of them. This correlates with the findings of a survey of medical doctors at the elite-level of men’s football who considered training load and wellness monitoring to be critical in mitigating the risk of injury [20]. Only a quarter of the medical personnel in our study considered musculoskeletal monitoring tests to be important tools in the prevention of injury, which contrasts with its importance to medical personnel in elite-level men’s football [20]. This may be due to the unavailability of medical staff at all training sessions in the WNL, as was suggested by one of the medical personnel, thus rendering it impossible to routinely undertake this type of monitoring. Alternatively, the medical personnel in our study may have agreed with the view of Bahr [63] that screening tests are unlikely to be able to predict the occurrence of injuries.

Bullock et al. [64] also highlighted the importance of following a rigorous methodology in the development and validation of clinical prediction models alongside transparent reporting of the findings in order to allow users to assess their potential usefulness. However, a recent systematic review of the methodological conduct and performance of existing musculoskeletal injury prediction models in sport reported that 98% of the models had a high risk of bias and none of them could be recommended for use in practice, thus highlighting the practical difficulties for practitioners working in sport [65].

Despite half of the players emphasising the importance of carefully designed injury surveillance systems in the construction of injury prevention programmes [66], only one of the head coaches mentioned this. This suggests that the leaders in many of the teams did not see the potential for the systematic collection of injury data to better inform their training practices, thus highlighting the challenges of implementing injury prevention practices in sport [67]. Some of the participants saw the results of the injury surveillance system as an opportunity to lobby for improved medical care support from governing bodies responsible for funding, thus highlighting the importance of getting “buy in” from all stakeholders in the protection of athlete health [68].

Almost all the coaches considered sub-maximal mini-band exercises to be effective in the prevention of injuries, despite an absence of evidence supporting this in the literature. This may be due to the visibility of these exercises during warm-ups in elite-level women’s and men’s football. Many of the coaches, medical personnel, and players considered training session modification to be an important injury prevention strategy based on players’ injury histories and match schedules. This highlights the importance of providing head coaches with more education and practical skills on training load monitoring and session design, including principles such as progressive overload and specificity during formal coach education licences so that they can adequately prepare players for the demands of match-play [69].

Several of the participants emphasised the use of jumping and landing exercises in the prevention of knee injuries and regular sprinting and high-speed running exposure in the prevention of muscle injuries, thus demonstrating an awareness of the evidence that supports these strategies [70–72]. However, none of the head coaches mentioned monitoring sprinting and high-speed running exposure. This is extremely concerning because they (coaches) have the primary role in training session design, and regular exposure to high-speed running and sprinting is an important injury risk mitigation strategy in football [72]. This may have had an influence on the high reported incidence rate of hamstring injuries in the WNL during the 2018 and 2019 seasons [6]. Some of the medical personnel and players also spoke about the importance of proprioceptive exercises in the prevention of ankle injuries and there is some evidence to support this although, in general, the quality of studies remains low [73, 74]. Prophylactic ankle taping was mentioned by two of the players and this has the best outcome in terms of cost and injury risk reduction [74], although the absence of medical personnel availability at all training sessions in the WNL was likely to have limited the implementation of a prophylactic ankle taping strategy.

The small number of participants, including only one medical personnel, who mentioned the use of the FIFA 11+, Nordic hamstring curls, isometric exercises and the Copenhagen adduction exercise suggests that either coaches, medical personnel and players in elite-level women’s football are not aware of the evidence base supporting their use in different football environments[70, 75–77] or that they do not consider them to be fit for purpose in the context of elite-level women’s football [78]. Lindblom et al. [79] presented some evidence from coaches in amateur-level senior women’s teams in Sweden of how they modified elements of the Knee Control injury prevention exercise programme to gain player buy-in. McCall et al. [80] undertook a Delphi survey of expert practitioners working in the big 5 male professional leagues in Europe to explore the perceived most effective exercise-based strategies to prevent muscle injuries in elite-level men’s footballers. A similar survey is needed in elite-level women’s football to provide a clearer insight into current practices.

### Knowledge, Attitudes, and Behaviours Towards Injury Management

Many of the players, coaches, and medical personnel believed that the use of student medical personnel in place of qualified practitioners and the unavailability of medical personnel at all training sessions and matches had a negative effect on the management of injuries in the WNL. It is possible that this may have led to many players playing with injuries as was identified in elite-level men’s football [81, 82]. Ardern et al. [83] emphasise the importance of high-quality intensive rehabilitation in successful return-to-sport (RTS) for athletes. A third of the participants believed that medical personnel needed to have a strong knowledge of RTS strategies. However, there is a dearth of evidence-based RTS criteria for many of the most common injuries in sport [15, 83], although Fältström et al. [84] used a classification and regression tree analysis involving functional performance, clinical assessment, and psychological factors, to accurately identify Swedish female footballers at high risk for a second ACL injury. This could be used to inform RTS decision-making. There are also some case examples of successful return to elite-level women’s football after severe injury using high quality clinical practice [85].

Several of the participants considered the interpersonal and communication skills of medical personnel to be important in the management of injuries. The consensus statement on RTS from injury [83], reinforces the importance of clear lines of communication between medical personnel, players and coaches during the RTS process—so the communication skills of medical personnel are critical. Significantly, some of the players voiced concerns over a lack of duty of care for them by medical personnel and this is at odds with the importance Thornton [86] places on the responsibility of medical personnel to create a supportive interpersonal environment for athletes, as well as Truong et al.’s [87] call for medical practitioners to take the social and contextual side of injury seriously. Without this support from medical personnel, players may choose not to self-report when they have an injury; this could lead to inaccurate recording of injury incidence rates and injury severity [88]. Moreover, safe and clear communication channels between staff and players are required to nurture healthy interpersonal relationships with players [89]. There was also a view that some of the medical personnel were not competent in the management of concussion. This is concerning, as careful management of concussed or suspected concussed athletes, including their immediate removal from sport, plays a vital role in protecting their health [90].

None of the participants mentioned any of the theoretical models [91–94] that exist to help the player, coach, and medical personnel in guiding the RTS decision-making process, although some of the participants discussed topics that are included in these models. For example, player injury history, player experience level, the type of injury, the time of the season and match importance all influenced the thinking of the participants in the RTS process.

Some of the players identified that inexperienced players were not given a voice in the RTS conversation with coaches and medical personnel, whereas more experienced players were included in the decision-making process. Loose et al. [95] highlighted big differences in the RTS beliefs between coaches and players in elite-level men’s football and suggest that without consensus between both groups, successful RTS is difficult. Dijkstra et al. [96] and Verhagen and Boling [97] suggest that a well organised injury management system should lead to the athlete being well informed enough on the injury to be able to be part of the RTS decision-making team. Some of the participants in our study did not think that inexperienced players were given a voice in those discussions, but there was also a view that players needed to trust that what they said to medical personnel would not affect team selection. Without a voice in conversations between medical personnel and coaches, it is unlikely that players will communicate openly with coaches and medical personnel. Vella et al. [88] also emphasised the importance of building trusting relationships between players, coaches, and medical personnel in relation to injury reporting in order to better protect the health of players. A more participative leadership style from coaches and better communication channels could lead to more player-specific injury management strategies with the potential benefit of lowering injury incidence rates, injury burden, and improving performance [10, 11, 68, 88]. To achieve this type of collaborative working environment in team sports, medical staff need to develop the required leadership skills that foster trusting relationships between them and the players and coaches that they work with [86, 98, 99].

### Methodological Considerations

In terms of rigor, selected principles (e.g., saturation, member participation validation, and pilot interviews) adhering to COREQ (Additional file 1) and more aligned procedures were employed [22, 32, 100]. Whilst randomly selecting participants may have produced different results and removed any potential selection bias, we employed data triangulation (i.e., athletes, medical personnel, coaches) in the collection of data to enhance credibility and representativeness [31].

Regarding rich rigour, the first author conducted all the interviews and his a priori knowledge of professional soccer and insider status as a former elite-athlete and Chartered Physiotherapist with elite-level athletes enabled access to and facilitated the establishment of rapport and trust with the participants, as evidenced by the collection of rich and highly sensitive data. The use of football-related terminology and meanings with which the interviewees were familiar enabled the participants to open-up and talk, facilitating the sharing of honest and, at times, sensitive information. For sincerity, the first author kept a reflexive journal designed to acknowledge his position[28, 32] by documenting his self-critical accounts of the research process to record personal reflections, challenges, and insights [29]. Numerous collaborative and reflexive meetings with the research team (DH, SK, MR, ED) were conducted throughout the research to enhance critical dialogue, challenge the analytical coding process, theme generation and naming, and to scrutinise and challenge the first authors (co)construction of knowledge and alternative interpretations of the data to ensure they were valid and grounded in the data[98] and to achieve richer interpretations of meaning [28, 32]. (Additional file 1).

For confirmability and dependability, and by providing neutrality to the findings [22], peer-triangulation was adopted during the coding process involving two coders (SK, MR) who had extensive experience of qualitative research. The aim was not about ‘achieving consensus between coders’ but concerned their collaborative ‘reflective and thoughtful engagement’ with the data and ‘the analytic process’ [28] to develop a richer more nuanced reading of the data [29]. Regarding researcher triangulation, and to enhance credibility, all the authors acted as critical friends [32] by engaging in a process of critical dialogue, which was designed to to challenge and scrutinise interpretations of the findings [31].

A limitation of these findings is that they are contextual and circumstantial. However, the utilisation of rich descriptions and visual figures allows the reader to judge the findings and our interpretations, thus also increasing dependability [100]. Unlike statistical generalisations, naturalistic generalisations [101] and transferable findings may be tentatively used to extrapolate to recent studies in elite-level women’s football [7]. Whilst we urge caution when making direct comparisons to women’s football in other countries and different levels of the game, the findings may resonate with employees in and researchers of elite-level women’s football and resemble readers’ experiences, settings they move in, events they’ve observed or heard about, and people they have talked to [101].

## Conclusion

The paucity of research in women’s football in comparison to the men’s game means that many of the beliefs of players, head coaches, and medical personnel in women’s football cannot be supported with empirical evidence. Many of the factors that have been proven to increase the risk of injury in elite-level men’s football, such as fixture congestion and strength deficits, were perceived by the players, head coaches, and medical personnel to also be risk factors for injury in women’s football, despite the lack of supporting evidence. Half of the participants also believed that menstrual cycle stage was a risk factor for injury although there is a dearth of evidence available to confirm these views. There was a lack of awareness amongst the majority of the participants of those risk factors for injury in women’s football that have some evidence to support them, such as previous injury, increased joint laxity, higher body mass index and trait anxiety. This highlights possible gaps in the education of the stakeholders participating in women’s football.

There was a good understanding of the importance of jumping and landing exercises in the prevention of knee injuries in particular amongst many of the participants, although evidence-based injury prevention exercises and programmes such as the Nordic hamstring curl, Copenhagen adduction and the FIFA 11 + were only mentioned by a small number of the participants. Also, hamstring muscle injuries have a high incidence rate in elite-level women’s football in Ireland and other leagues, yet none of the head coaches in our study discussed monitoring sprinting exposure during training—suggesting that players may have been inadequately prepared for match-play.

Whilst some of the players and coaches were happy with the quality of medical care available to their teams, overall dissatisfaction with medical care and S & C support was a consistent theme amongst the players in the study, with poor communication practices reported between players, medical personnel and head coaches. This was considered by many of the players to be a barrier to the implementation of effective injury risk reduction strategies and successful RTS practices. Player confidence in the support systems available to them is likely to be a critical element of successful teams and without this women’s football worldwide will not be able to continue to develop at the rate the players deserve.

### Perspective

More original research is required in elite-level women’s football exploring risk factors for injury, injury prevention initiatives and contextual RTS strategies so that players, head coaches, and medical personnel can use evidence that is both up-to-date and specific to their environment. Players need to have access to qualified and experienced medical and S & C personnel who have the skills and personalities to create a collaborative high-performance environment in unison with the coaching staff. Without this, elite-level women football players will continue to be exposed to unacceptable risks to their health where injuries are not managed to the best possible standard.

## Supplementary Information

**Additional file 1.** Consolidated criteria for reporting qualitative studies: 32-item checklist

**Additional file 2.** Player, head coaches, and medical personnel interview guide

**Additional file 3.** Example Quotes supporting the Theme “Injuries” and its associated Sub-themes. All quotes are categorised according to the three domains of:knowledge;attitudes;practices. C = Head Coaches; M = Medical Personnel; P = Players

**Additional file 4.** Example Quotes supporting the Theme “Prevention of Injuries” and its associated Sub-themes. All quotes are categorised according to the three domains of:knowledge;attitudes;practices. C = Head Coaches; M = Medical Personnel; P = Players

**Additional file 5.** Example Quotes supporting the Theme “Injury Management” and its associated Sub-themes. All quotes are categorised according to the three domains of:knowledge;attitudes;practices. C = Head Coaches; M = Medical Personnel; P = Players

**Additional file 6.** NVIVO coding

## Data Availability

Due to the potential for selective disclosure, the verbatim transcripts are unavailable. All data relevant to the study are included in the article.

## Abbreviations

WNL: Women’s National League
S & C: Strength and conditioning
RTS: Return-to-sport
ACL: Anterior cruciate ligament
MCL: Medial collateral ligament

## References

[1] Federation Internationale de Football Association (FIFA). The vision 2020–2023. 2020. https://publications.fifa.com/en/vision-report-2021/. Accessed 20 June 2022

[2] Union of European Football Associations (UEFA). Time for action: UEFA strategy 2019–2024. 2019. https://www.uefa.com/MultimediaFiles/Download/uefaorg/Womensfootball/02/60/51/38/2605138. Accessed 12 June 2022

[3] Vescovi JD, Fernandes E, Klas A (2021). Physical Demands of women’s soccer matches: a perspective across the developmental spectrum. Front Sports Act Living.

[4] Bradley P, Scott D. Physical Analysis of the FIFA Women’s World Cup France 2019TM. 2020; FIFA, Zurich.

[5] Meeuwisse WH, Tyreman H, Hagel B, Emery C (2007). A dynamic model of etiology in sport injury: the recursive nature of risk and causation. Clin J Sport Med.

[6] Horan D, Blake C, Hägglund M (2022). Injuries in elite-level women's football-a two-year prospective study in the Irish Women's National League. Scand J Med Sci Sports.

[7] Larruskain J, Lekue JA, Diaz N, Odriozola A, Gil SM (2018). A comparison of injuries in elite male and female football players: a five-season prospective study. Scand J Med Sci Sport.

[8] Hägglund M, Waldén M, Ekstrand J (2009). Injuries among male and female elite football players. Scand J Med Sci Sport.

[9] Geertsema C, Geertsema L, Farooq A, Harøy J, Oester C, Weber A (2021). Injury prevention knowledge, beliefs and strategies in elite female footballers at the FIFA Women’s World Cup France 2019. Br J Sports Med.

[10] Ekstrand J, Lundqvist D, Lagerbäck L, Vouillamoz M, Papadimitiou N, Karlsson J (2018). Is there a correlation between coaches' leadership styles and injuries in elite football teams? A study of 36 elite teams in 17 countries. Br J Sports Med.

[11] Ekstrand J, Lundqvist D, Davison M, D'Hooghe M, Pensgaard AM (2019). Communication quality between the medical team and the head coach/manager is associated with injury burden and player availability in elite football clubs. Br J Sports Med.

[12] Finch C (2006). A new framework for research leading to sports injury prevention. J Sci Med Sport.

[13] Bolling C, Van MW, Pasman HR, Verhagen E (2018). Context matters : revisiting the first step of the ‘sequence of prevention’ of sports injuries. Sport Med.

[14] Verhagen E, Voogt N, Bruinsma A, Finch CF (2014). A knowledge transfer scheme to bridge the gap between science and practice: an integration of existing research frameworks into a tool for practice. Br J Sports Med.

[15] Okholm Kryger K, Wang A, Mehta R, Impellizzeri F, Massey A, Harrison M, Glendinning R, McCall A (2022). Can we evidence-base injury prevention and management in women's football? A scoping review. Res Sports Med..

[16] Ekegren C, Gabbe B, Finch C (2016). Sports injury surveillance systems: a review of methods and data quality. Sports Med.

[17] O’Brien J, Donaldson A, Finch CF (2016). It will take more than an existing exercise programme to prevent injury. Br J Sports Med.

[18] Verhagen E, Bolling C (2018). We dare to ask new questions. Are we also brave enough to change our approaches?. Transl Sports Med..

[19] Verhagen E, van Stralen MM, van Mechelen W (2010). Behaviour, the key factor for sports injury prevention. Sport Med.

[20] McCall A, Dupont G, Ekstrand J (2016). Injury prevention strategies, coach compliance and player adherence of 33 of the UEFA elite Club injury study teams: a survey of teams' head medical officers. Br J Sports Med.

[21] Bekker S, Bolling C, Ahmed OH, Badenhorst M, Carmichael J, Fagher K (2020). Athlete health protection: why qualitative research matters. J Sci Med Sport..

[22] Bolling C, Delfino Barboza S, van Mechelen W, Pasman HR (2020). Letting the cat out of the bag: athletes, coaches and physiotherapists share their perspectives on injury prevention in elite sports. Br J Sports Med.

[23] Sandberg J (2005). How do we justify knowledge produced within interpretive approaches?. Organ Res Methods.

[24] Schwandt TA, Denzin N, Lincoln Y (1994). Constructivist, interpretivist approaches to human inquiry. Handbook of qualitative research. The Landscape of qualitative research: theories and issues.

[25] Tong A, Sainsbury P, Craig J (2007). Consolidated criteria for reporting qualitative research (COREQ): a 32-item checklist for interviews and focus groups. Int J Qual Health Care.

[26] Bonell Monsonís O, Verhagen E, Kaux JF, Bolling C (2021). 'I always considered I needed injury prevention to become an elite athlete': the road to the Olympics from the athlete and staff perspective. BMJ Open Sport Exerc Med.

[27] Alvesson M, Sandberg J (2011). Generating research questions through problematization. Acad Manag Rev.

[28] Braun V, Clarke V (2019). Reflecting on reflexive thematic analysis. Qual Res Sport Exerc Health.

[29] Braun V, Clarke V (2021). One size fits all? What counts as quality practice in (reflexive) thematic analysis?. Qual Res Psychol.

[30] Smith BM, Sparkes AC (2019). Routledge handbook of qualitative research in sport and exercise.

[31] Skinner J, Edwards A, Smith AC (2021). Qualitative research in sport management.

[32] Smith B, McGannon KR (2017). Developing rigor in qualitative research: Problems and opportunities within sport and exercise psychology. Int Rev Sport Exerc Psychol.

[33] Waldén M, Hägglund M, Magnusson H, Ekstrand J (2011). Anterior cruciate ligament injury in elite football: a prospective three-cohort study. Knee Surg Sports Traumatol Arthrosc.

[34] Waldén M, Hägglund M, Ekstrand J (2007). Football injuries during European Championships 2004–2005. Knee Surg Sports Traumatol Arthrosc.

[35] Eirale C, Tol JL, Farooq A, Smiley F, Chalabi H (2013). Low injury rate strongly correlates with team success in Qatari professional football. Br J Sports Med.

[36] Hägglund M, Waldén M, Magnusson H, Kristenson K, Bengtsson H, Ekstrand J (2013). Injuries affect team performance negatively in professional football: an 11-year follow-up of the UEFA Champions League injury study. Br J Sports Med.

[37] Martin D, Timmins K, Cowie C, Alty J, Mehta R, Tang A (2021). Injury incidence across the menstrual cycle in international footballers. Front Sports Act Living.

[38] Bruinvels G, Burden RJ, McGregor AJ, Ackerman KE, Dooley M, Richards T (2017). Sport, exercise and the menstrual cycle: where is the research?. Br J Sports Med.

[39] Bruinvels G, Goldsmith E, Blagrove R, Simpkin A, Lewis N, Morton K (2021). Prevalence and frequency of menstrual cycle symptoms are associated with availability to train and compete: a study of 6812 exercising women recruited using the Strava exercise app. Br J Sports Med.

[40] Carling C, McCall A, Le Gall F, Dupont G (2016). The impact of short periods of match congestion on injury risk and patterns in an elite football club. Br J Sports Med.

[41] Bengtsson H, Ekstrand J, Hägglund M (2013). Muscle injury rates in professional football increase with fixture congestion: an 11-year follow-up of the UEFA Champions League injury study. Br J Sports Med.

[42] Dellal A, Lago-Peñas C, Rey E, Chamari K, Orhant E (2015). The effects of a congested fixture period on physical performance, technical activity and injury rate during matches in a professional soccer team. Br J Sports Med.

[43] Parsons JL, Coen SE, Bekker S (2021). Anterior cruciate ligament injury: towards a gendered environmental approach. Br J Sports Med.

[44] Lauersen JB, Andersen TE, Andersen LB (2018). Strength training as superior, dose-dependent and safe prevention of acute and overuse sports injuries: a systematic review, qualitative analysis and meta-analysis. Br J Sports Med.

[45] Fanchini M, Steendahl IB, Impellizzeri FM, Pruna R, Dupont G, Coutts AJ (2020). Exercise-based strategies to prevent muscle injury in elite footballers: a systematic review and best evidence synthesis. Sports Med.

[46] Gabbett TJ, Kennelly S, Sheehan J, Hawkins R, Milsom J, King E (2016). If overuse injury is a 'training load error', should undertraining be viewed the same way?. Br J Sports Med.

[47] Gabbett TJ (2016). The training-injury prevention paradox: should athletes be training smarter and harder?. Br J Sports Med.

[48] Drew MK, Finch CF (2016). The relationship between training load and injury, illness and soreness: a systematic and literature review. Sports Med.

[49] Kalkhoven JT, Watsford ML, Coutts AJ, Edwards WB, Impellizzeri FM (2021). Training load and injury: causal pathways and future directions. Sports Med.

[50] Costa JA, Rago V, Brito P, Figueiredo P, Sousa A, Abade E (2022). Training in women soccer players: a systematic review on training load monitoring. Front Psychol.

[51] Xiao M, Nguyen JN, Hwang CE, Abrams GD (2021). increased lower extremity injury risk associated with player load and distance in collegiate women's soccer. Orthop J Sports Med.

[52] Impellizzeri FM, McCall A, Ward P, Bornn L, Coutts AJ (2020). Training load and its role in injury prevention, part 2: conceptual and methodologic pitfalls. J Athl Train.

[53] Harkness-Armstrong A, Till K, Datson N, Myhill N, Emmonds S (2022). A systematic review of match-play characteristics in women's soccer. PLoS ONE.

[54] Union of European Football Associations (UEFA). Together for the future of football: UEFA strategy 2019–2024. 2019. https://editorial.uefa.com/resources/026e-1389dc45d12e-bdf69579a884-1000/together_for_the_future_of_football.pdf. Accessed 12 June 2022

[55] Fältström A, Kvist J, Gauffin H, Hägglund M (2019). Female soccer players with anterior cruciate ligament reconstruction have a higher risk of new knee injuries and quit soccer to a higher degree than knee-healthy controls. Am J Sports Med.

[56] Faude O, Junge A, Kindermann W, Dvorak J (2006). Risk factors for injuries in elite female soccer players. Br J Sports Med.

[57] Söderman K, Alfredson H, Pietilä T, Werner S (2001). Risk factors for leg injuries in female soccer players: a prospective investigation during one out-door season. Knee Surg Sports Traumatol Arthrosc.

[58] Nilstad A, Andersen TE, Bahr R, Holme I, Steffen K (2014). Risk factors for lower extremity injuries in elite female soccer players. Am J Sports Med.

[59] Ostenberg A, Roos H (2000). Injury risk factors in female European football. A prospective study of 123 players during one season. Scand J Med Sci Sports..

[60] Ivarsson A, Johnson U, Podlog L (2013). Psychological predictors of injury occurrence: a prospective investigation of professional Swedish soccer players. J Sport Rehabil.

[61] Ivarsson A, Johnson U, Karlsson J, Börjesson M, Hägglund M, Andersen M, Waldén M (2019). Elite female footballers’ stories of sociocultural factors, emotions, and behaviours prior to anterior cruciate ligament injury. Int J Sport Exerc Psychol.

[62] Pensgaard AM, Ivarsson A, Nilstad A, Solstad BE, Steffen K (2018). Psychosocial stress factors, including the relationship with the coach, and their influence on acute and overuse injury risk in elite female football players. BMJ Open Sport Exerc Med.

[63] Bahr R (2016). Why screening tests to predict injury do not work—and probably never will…: a critical review. Br J Sports Med.

[64] Bullock GS, Hughes T, Sergeant JC, Callaghan MJ, Riley R, Collins G (2021). Methods matter: clinical prediction models will benefit sports medicine practice, but only if they are properly developed and validated. Br J Sports Med.

[65] Bullock GS, Hughes T, Arundale AH, Ward P, Collins GS, Kluzek S (2022). Black box prediction methods in sports medicine deserve a red card for reckless practice: a change of tactics is needed to advance athlete care. Sports Med.

[66] Bahr R, Clarsen B, Derman W, Dvorak J, Emery CA, Finch CF (2020). International Olympic Committee Consensus Statement: methods for recording and reporting of epidemiological data on injury and illness in sports 2020 (including the STROBE extension for sports injury and illness surveillance (STROBE-SIIS)). Orthop J Sports Med..

[67] Donaldson A, Finch CF (2013). Applying implementation science to sports injury prevention. Br J Sports Med.

[68] Drew MK, Cook J, Finch CF (2016). Sports-related workload and injury risk: simply knowing the risks will not prevent injuries: narrative review. Br J Sports Med.

[69] Klein C, Henke T, Luig P, Platen P (2018). Leaving injury prevention theoretical? Ask the coach!—A survey of 1012 football coaches in Germany. Ger J Exerc Sport Res.

[70] Crossley KM, Patterson BE, Culvenor AG, Bruder AM, Mosler AB, Mentiplay BF (2020). Making football safer for women: a systematic review and meta-analysis of injury prevention programmes in 11 773 female football (soccer) players. Br J Sports Med.

[71] Arundale AJH, Bizzini M, Giordano A, Hewett TE, Logerstedt DS, Mandelbaum B (2018). Exercise-based knee and anterior cruciate ligament injury prevention. J Orthop Sports Phys Ther.

[72] Malone S, Owen A, Mendes B, Hughes B, Collins K, Gabbett TJ (2018). High-speed running and sprinting as an injury risk factor in soccer: Can well-developed physical qualities reduce the risk?. J Sci Med Sport.

[73] Caldemeyer LE, Brown SM, Mulcahey MK (2020). Neuromuscular training for the prevention of ankle sprains in female athletes: a systematic review. Phys Sportsmed.

[74] Kaminski TW, Needle AR, Delahunt E (2019). Prevention of lateral ankle sprains. J Athl Train.

[75] Alahmad TA, Kearney P, Cahalan R (2020). Injury in elite women's soccer: a systematic review. Phys Sportsmed.

[76] van Dyk N, Behan FP, Whiteley R (2019). Including the Nordic hamstring exercise in injury prevention programmes halves the rate of hamstring injuries: a systematic review and meta-analysis of 8459 athletes. Br J Sports Med.

[77] Ishøi L, Thorborg K (2021). Copenhagen adduction exercise can increase eccentric strength and mitigate the risk of groin problems: but how much is enough!. Br J Sports Med.

[78] Buchheit M, Eirale C, Simpson BM, Lacome M (2019). Injury rate and prevention in elite football: let us first search within our own hearts. Br J Sports Med.

[79] Lindblom H, Carlfjord S, Hägglund M (2018). Adoption and use of an injury prevention exercise program in female football: a qualitative study among coaches. Scand J Med Sci Sports.

[80] McCall A, Pruna R, Van der Horst N, Dupont G, Buchheit M, Coutts AJ (2020). Exercise-based strategies to prevent muscle injury in male elite footballers: an expert-led Delphi survey of 21 practitioners belonging to 18 teams from the big-5 European leagues. Sports Med.

[81] Hammond LE, Lilley JM, Pope GD, Ribbans WJ (2014). The impact of playing in matches while injured on injury surveillance findings in professional football. Scand J Med Sci Sports.

[82] Roderick M (2006). Adding insult to injury: workplace injury in English professional football. Sociol Health Illn.

[83] Ardern CL, Glasgow P, Schneiders A, Witvrouw E, Clarsen B, Cools A (2016). 2016 Consensus statement on return to sport from the First World Congress in Sports Physical Therapy. Bern Br J Sports Med.

[84] Fältström A, Kvist J, Bittencourt NFN, Mendonça LD, Hägglund M (2021). Clinical risk profile for a second anterior cruciate ligament injury in female soccer players after anterior cruciate ligament reconstruction. Am J Sports Med.

[85] Taberner M, van Dyk N, Allen T, Jain N, Richter C, Drust B (2020). Physical preparation and return to performance of an elite female football player following ACL reconstruction: a journey to the FIFA Women's World Cup. BMJ Open Sport Exerc Med.

[86] Thornton JS (2020). Athlete autonomy, supportive interpersonal environments and clinicians' duty of care; as leaders in sport and sports medicine, the onus is on us: the clinicians. Br J Sports Med.

[87] Truong LK, Bekker S, Whittaker JL (2021). Removing the training wheels: embracing the social, contextual and psychological in sports medicine. Br J Sports Med.

[88] Vella S, Bolling C, Verhagen E, Moore IS (2021). Perceiving, reporting and managing an injury—perspectives from national team football players, coaches, and health professionals. Sci Med Footb.

[89] Burns L, Weissensteiner JR, Cohen M (2019). Supportive interpersonal relationships: a key component to high-performance sport. Br J Sports Med.

[90] McCrory P, Meeuwisse W, Dvorak J, Aubry M, Bailes J, Broglio S (2017). Consensus statement on concussion in sport—the 5th international conference on concussion in sport held in Berlin, October 2016. Br J Sports Med.

[91] Shrier I (2015). Strategic Assessment of risk and risk tolerance (StARRT) framework for return-to-play decision-making. Br J Sports Med.

[92] Atkins E, Colville G, John M (2012). A ‘biopsychosocial’ model for recovery: a grounded theory study of families’ journeys after a Paediatric Intensive Care admission. Intensive Crit Care Nurs.

[93] Wiese-Bjornstal DM, Smith AM, Shaffer SM, Morrey M (1998). An integrated model of response to sport injury: psychological and sociological dynamics. J Appl Sport Psychol..

[94] Gabbett TJ, Hulin BT, Blanch P, Whiteley R (2016). High training workloads alone do not cause sports injuries: how you get there is the key issue. Br J Sports Med.

[95] Loose O, Achenbach L, Fellner B, Lehmann J, Jansen P, Nerlich M (2018). Injury prevention and return to play strategies in elite football: no consent between players and team coaches. Arch Orthop Trauma Surg.

[96] Dijkstra HP, Pollock N, Chakraverty R, Ardern CL (2017). Return to play in elite sport: a shared decision-making process. Br J Sports Med.

[97] Verhagen E, Bolling C (2015). Protecting the health of the @hlete: how online technology may aid our common goal to prevent injury and illness in sport. Br J Sports Med.

[98] Tayne S, Hutchinson MR, O'Connor FG, Taylor DC, Musahl V, Indelicato P (2020). Leadership for the team physician. Curr Sports Med Rep.

[99] Verhagen E, Mellette J, Konin J, Scott R, Brito J, McCall A (2020). Taking the lead towards healthy performance: the requirement of leadership to elevate the health and performance teams in elite sports. BMJ Open Sport Exerc Med.

[100] Tracy SJ (2010). Qualitative quality: Eight “big-tent” criteria for excellent qualitative research. Qual Inq.

[101] Smith B (2018). Generalizability in qualitative research: misunderstandings, opportunities and recommendations for the sport and exercise sciences. Qual Res Sport Exerc Health.

