# Supplementary material for: Players’, Head Coaches', And Medical Personnels' Knowledge, Understandings and Perceptions of Injuries and Injury Prevention in Elite-Level Women’s Football in Ireland
Source: Sports Med Open. 2023 Jul 29;9:64. doi: 10.1186/s40798-023-00603-6 (PMC10387024; doi:10.1186/s40798-023-00603-6)
Supplement: Supplementary file 1 — Additional file 1. Consolidated criteria for reporting qualitative studies: 32-item checklist [file 40798_2023_603_MOESM1_ESM.docx]

**Manuscript Title:** Players’, head coaches and medical personnel knowledge, understandings, and perceptions of injuries and injury prevention in elite-level women’s football in Ireland.

**Journal:** Sports Medicine Open

**Authors:** Dan Horan,^1,5^ Seamus Kelly,^1^ Martin Hägglund,^2,3^ Catherine Blake,^1^ Mark Roe,^1^ Eamonn Delahunt.^1,4^

**Authors’ Affiliations:**

^1^ School of Public Health, Physiotherapy and Sports Science, University College Dublin, Dublin, Ireland

^2^ Football Research Group, Linköping University, Linköping, Sweden

^3^ Division of Physiotherapy, Department of Health, Medicine and Caring Sciences, Linköping University, Linköping, Sweden

^4^ Institute for Sport and Health, University College Dublin, Dublin, Ireland

^5^ Department of Sport, Leisure & Childhood Studies, Munster Technological University, Cork, Ireland

**Corresponding Author Email Address**

Dan Horan: danhoran10@gmail.com

**Consolidated criteria for reporting qualitative studies (COREQ): 32-item checklist**

Developed from:

Tong A, Sainsbury P, Craig J. Consolidated criteria for reporting qualitative research (COREQ): a 32-item checklist for interviews and focus groups. *International Journal for Quality in Health Care*. 2007;19(6):349 – 357

| **No. Item** | **Guide questions/description** |  | **Reported on Page #** |
| --- | --- | --- | --- |
| **Domain 1: Research team and reﬂexivity** |  |  |  |
| *Personal Characteristics* |  |  |  |
| 1. Inter viewer/facilitator | Which author/s conducted the interview or focus group? | Dan Horan conducted all the interviews. | Reported, Page 7 |
| 2. Credentials | What were the researcher’s credentials? E.g., PhD, MD | Bachelor of Commerce Degree, University College Dublin. MSc Administrative Studies, Boston College. MSc Exercise and Health Sciences, University of Bristol. MSc Physiotherapy, Queen Margaret University, Edinburgh. | Not reported |
| 3. Occupation | What was their occupation at the time of the study? | Part-time PhD Researcher, Chartered Physiotherapist, Head of Research at the Football Association of Ireland | Not reported |
| 4. Gender | Was the researcher male or female? | Male | Not reported |
| 5. Experience and training | What experience or training did the researcher have? | He has published qualitative research and additional manuscripts are currently undergoing peer-review. As part of his PhD in UCD, he completed training in advanced qualitative analysis, knowledge synthesis, systematic reviews, and meta-analysis. | Not reported |
| *Relationship with participants* |  |  |  |
| 6. Relationship established | Was a relationship established prior to study commencement? | Some Participants (Players: n=12; head coaches: N=6; medical personnel: N=2) were known to the first author through his experience working as a physiotherapist for numerous teams in Ireland and through their involvement in previous research. The remainder (n=12) were not known to the researcher prior to being contacted and subsequently interviewed. | Not reported |
| 7. Participant knowledge of the interviewer | What did the participants know about the researcher? e.g. personal goals, reasons for doing the research | All participants were aware that the researcher was a PhD student at University College Dublin. All participants received an information letter by email outlining a general overview of the research. | Not reported |
| 8. Interviewer characteristics | What characteristics were reported about the inter viewer/facilitator? e.g., Bias, assumptions, reasons, and interests in the research topic | Interviewer was a Chartered Physiotherapist with extensive experience in elite-level and international football and was Head of Research at the Football Association of Ireland. | Not reported |

| **Domain 2: study design** |  |  |  |
| --- | --- | --- | --- |
| *Theoretical framework* |  |  |  |
| 9. Methodological orientation and Theory | What methodological orientation was stated to underpin the study? e.g., grounded theory, discourse analysis, ethnography, phenomenology, content analysis | This study is located within an interpretive, constructivist research paradigm. This paradigm assumes that reality exists in the form of multiple individuals’ constructions about the world which are shaped through lived experiences and that there is no single external reality of the individual. Unlike more descriptive or positivist approaches, a more interpretivist, constructivist approach was adopted which places more emphasis on inductive exploration and where meaning and experience are socially produced and reproduced. Our overall motivation for adopting this methodological approach is for discovering meaning and understanding the participants’ lived experiences in context [1, 2]. | Not reported |
| *Participant selection* |  |  |  |
| 10. Sampling | How were participants selected? e.g. purposive, convenience, consecutive, snowball | Non-probability sampling techniques, or purposive sampling, often referred to as “judgement samples” was adopted where participants were subjectively recruited [3] based on the personal judgement of, and numerous discussions between, the research team (DH, SK, ED). Rather than breadth, our aim was to recruit participants based on relevance and variations in the participants in terms of their demographics, characteristics, and experiences [3]. Consequently, participants were selected that could provide more relevant data and a depended understanding based on their ‘connection to’ and ‘involvement in’ the research topic [3]. We acknowledge that non-random samples may lack representativeness, are non-probabilistic, lack statistical power and may lack generalisability [3]. However, the sample limitations may be offset by the highly specialised nature of the participants and their knowledge and experience with the current research topic. Moreover, the sample was subjected to extensive and in-depth data examination leading to a greater level of understanding of the research aims. | Not reported |
| 11. Method of approach | How were participants approached? e.g., face-to-face, telephone, mail, email | All participants received an introductory telephone call and email from the primary researcher (DH), that provided an initial overview of the study and an invitation to participate. Participants were also provided with contact details should they wish to seek further information. | Not reported |
| 12. Sample size | How many participants were in the study? | Determining the “correct size” of a qualitative sample is a contentious issue in qualitative research [3]. Our goal was to capture variation, or breadth, across participants and we argue that the sample size provides reasonable coverage [3]. Rich, in-depth data was collected from information-rich participants. The total number of participants was deemed appropriate based on attaining data and meaning saturation [4, 5]. | Not reported |
| 13. Non-participation | How many people refused to participate or dropped out? Reasons? | One of the contacted participants did not participate in the research due to their unavailability. | Not reported |
| *Setting* |  |  |  |
| 14. Setting of data collection | Where was the data collected? e.g. home, clinic, workplace | Due to COVID-19, 28 of the interviews were video-based and took place online (Zoom/Teams). 4 took place face-to-face at a location convenient to the participants. | Not reported |
| 15. Presence of non-participants | Was anyone else present besides the participants and researchers? | No. | Not reported |
| 16. Description of sample | What are the important characteristics of the sample? e.g. demographic data, date | Criterion-based techniques were adopted to purposively recruit a heterogenous sample of elite head coaches, elite female athletes, and medical staff representing clubs in the Women’s National League (WNL) in Ireland. Specifically, the inclusion criteria were that the participants, at the time of the interview, had to have experience of the WNL in Ireland.  All the athletes had various levels of playing experience with a WNL team. Some of the athletes had represented the national team at senior or underage level (i.e., under 17/under 19) and some were non-international-level athletes. Athletes also had various alternative careers or educational commitments.  All the medical personnel possessed experience of working with a WNL team. The medical and sport sciences support staff is heterogeneous among clubs and is largely dependent upon the financial resources of the clubs.  All the coaches had experience of managing a WNL team. The length of tenure varied with some head coaches having managed for a considerable number of years in the WNL. Some demographic information has been withheld to protect their anonymity and to avoid any potential for deductive disclosure due to the small number of head coaches. | Not reported |
| *Data collection* |  |  |  |
| 17. Interview guide | Were questions, prompts, guides provided by the authors? Was it pilot tested? | Semi-structured interviews are appropriate when exploring participants understanding and interpretation of the specific research questions being discussed [3]. A semi-structured interview guide (Supplementary file 2) was developed through a process of gap spotting [6, 7] and theoretical and pragmatic problematisation [8] of the literature (e.g., injury management and prevention, health protection) to bridge the gap between research and practice.  The interview guide was also informed by the researchers experience within the filed, further developed through discussions with the research team (ED, DH, MR,SK) and subsequently refined to enhance rigour after a pilot interview (not included in the study) with a coach that had considerable experience in the WNL. | Reported, Page 6.  Not reported |
| 18. Repeat interviews | Were repeat inter views carried out? If yes, how many? | No | Not reported |
| 19. Audio/visual recording | Did the research use audio or visual recording to collect the data? | All the interviews were recorded using a Dictaphone and laptop audio recorder software. Due to COVID-19, 28 of the interviews were video-based and took place online (i.e., Zoom/teams). While in-person interviews are often viewed as marginally superior to video-based interviews, participants in video-based interviews often divulge more personal details and there are limited restrictions in developing rapport or disclosing sensitive issues [9]. | Not reported |
| 20. Field notes | Were ﬁeld notes made during and/or after the interview or focus group? | Yes, the first author maintained a reflective journal throughout the study. | Not reported. |
| 21. Duration | What was the duration of the inter views or focus group? | The average length of the interviews was 47 minutes (range 28-111 minutes). | Reported, Page 7. |
| 22. Data saturation | Was data saturation discussed? | Data and meaning saturation identified in-text and was attained reached [4, 5]. Saturation, or informational redundancy, was reached where data collected from additional subjects would not contribute any new information [3]. | Not reported |
| 23. Transcripts returned | Were transcripts returned to participants for comment and/or correction? | The transcripts were transcribed verbatim, returned to all the participants and they were invited to provide any comments or reflections. No modifications were made, or feedback provided by the participants. Consequently, the adoption of member participant validation ensured that the meanings of the statements spoken during the interviews were accurately transcribed by the researcher, thereby enhancing the conformability and credibility of the data [5]. | Not reported |
| **Domain 3: analysis and ﬁndings** |  |  |  |
| *Data analysis* |  |  |  |
| 24. Number of data coders | How many data coders coded the data? | In the early stages of data analysis, SK independently open-coded a sample (n=6) of transcripts to sense-check how DH was coding the data, to compare initial insights generated using memos, discuss connections between the codes and themes, and to explore alternative interpretations of the data. After data collection ceased, SK and MR independently open-coded a separate sample (n=6) of transcripts. SK and MR possessed no medical experience but are experienced qualitative researchers and SK has experience at various coaching and playing levels of professional and semi-professional soccer. Minor discrepancies in the latent codes and theme naming were discussed. Overall, this double review involved discussion of the coding process, and the generation of main themes, and sub-themes. Then, SK, DH and ED discussed the findings over two meetings and the themes and any discrepancies in the naming of the themes were discussed. | Not reported |
| 25. Description of the coding tree | Did authors provide a description of the coding tree? | Detailed description of reflective thematic analysis provided, combined with visual representation of the codes and themes in the appendices. | Reported, Page 7. |
| 26. Derivation of themes | Were themes identiﬁed in advance or derived from the data? | Theme and sub-themes derived from data. | Reported, Page 7. |
| 27. Software | What software, if applicable, was used to manage the data? | The qualitative analysis software (QSR NVIVO-12) assisted in storing, structuring, and organising the data into thematic hierarchies. | Not reported |
| 28. Participant checking | Did participants provide feedback on the ﬁndings? | Providing feedback on the findings with the participants was not completed because of methodological and pragmatic challenges [10, 5] | Not reported |
| *Reporting* |  |  |  |
| 29. Quotations presented | Were participant quotations presented to illustrate the themes/ﬁndings? Was each quotation identiﬁed? e.g. participant number | Quotations from a range of different participants are used to illustrate themes and findings in-text. Further quotations provided in appendices. All quotations are identified by participant number. | Reported,  Page 9. |
| 30. Data and ﬁndings consistent | Was there consistency between the data presented and the ﬁndings? | Consistency between data presented and the study findings was achieved. | Reported, Page 9. |
| 31. Clarity of major themes | Were major themes clearly presented in the ﬁndings? | The main themes are derived from the data. Themes are clearly presented in-text and supporting documentation is included in the appendices. | Reported, Page 9. |
| 32. Clarity of minor themes | Is there a description of diverse cases or discussion of minor themes? | The minor themes are derived from the data. Minor themes are clearly presented in-text. Supporting documentation is included in the appendices. | Reported, Page 9. |

**References**

1. Sandberg, J. How do we justify knowledge produced within interpretive approaches? Organizational research methods. 2005;8(1):41-68.
2. Schwandt, T. A. Constructivist, interpretivist approaches to human inquiry. Handbook of qualitative research, 1(1994). In Denzin, N. and Lincoln, Y (Eds.), The Landscape of Qualitative Research: Theories and issues. (pp. 221-259). Thousand Oaks, CA: Sage Publications.
3. Skinner, J., Edwards, A., & Smith, A. C. (Eds.). Qualitative research in sport management (2nd Edition). 2021. London: Routledge.
4. Braun, V., & Clarke, V. Reflecting on reflexive thematic analysis. Qualitative research in sport, exercise and health. 2019;11(4):589-597.
5. Smith, B., & McGannon, K. R. Developing rigor in qualitative research: Problems and opportunities within sport and exercise psychology. International review of sport and exercise psychology. 2017;11(1):101-121.
6. Bolling C, Delfino Barboza S, van Mechelen W, Pasman HR. Letting the cat out of the bag: athletes, coaches and physiotherapists share their perspectives on injury prevention in elite sports. Br J Sports Med. 2020;54(14):871-877.
7. Bekker, S, Bolling, C, H Ahmed, O, Badenhorst M, Carmichael J, Fagher K, et al. Athlete health protection: why qualitative research matters. J Sci Med Sport. 2020;23(10):898–901
8. Alvesson, M., & Sandberg, J. Generating research questions through problematization. Academy of management review. 2011;36(2):247-271
9. Howlett M. Looking at the 'field' through a Zoom lens: Methodological reflections on conducting online research during a global pandemic. Qual Res. 2022;22(3):387-402.
10. Tracy, S. J. Qualitative quality: Eight “big-tent” criteria for excellent qualitative research. Qualitative inquiry. 2010;16(10):837-851.
