# Supplementary material for: Players’, Head Coaches', And Medical Personnels' Knowledge, Understandings and Perceptions of Injuries and Injury Prevention in Elite-Level Women’s Football in Ireland
Source: Sports Med Open. 2023 Jul 29;9:64. doi: 10.1186/s40798-023-00603-6 (PMC10387024; doi:10.1186/s40798-023-00603-6)
Supplement: Supplementary file 2 — Additional file 2. Player, head coaches, and medical personnel interview guide [file 40798_2023_603_MOESM2_ESM.docx]

**Manuscript Title:** Players’, head coaches and medical personnel knowledge, understandings, and perceptions of injuries and injury prevention in elite-level women’s football in Ireland.

**Journal:** Sports Medicine Open

**Authors:** Dan Horan,^1,5^ Seamus Kelly,^1^ Martin Hägglund,^2,3^ Catherine Blake,^1^ Mark Roe,^1^ Eamonn Delahunt.^1,4^

**Authors’ Affiliations:**

^1^ School of Public Health, Physiotherapy and Sports Science, University College Dublin, Dublin, Ireland

^2^ Football Research Group, Linköping University, Linköping, Sweden

^3^ Division of Physiotherapy, Department of Health, Medicine and Caring Sciences, Linköping University, Linköping, Sweden

^4^ Institute for Sport and Health, University College Dublin, Dublin, Ireland

^5^ Department of Sport, Leisure & Childhood Studies, Munster Technological University, Cork, Ireland

**Corresponding Author Email Address**

Dan Horan: danhoran10@gmail.com

**Player interview guide**

1. How do you know you are ready to perform in a game?

2. How do you know you are not ready to perform in a game?

3. (a) What information informs these decisions?

(b) What do you use to measure match performance?

(c) What are your key performance indicators?

4. Who decides if players are available to play?

5. (a) What strategies are used to ensure players are available to play?

(b) Who or what influences these strategies?

6. What are the core competencies required of medical personnel working in elite-level women’s football?

7. What are the core competencies required of strength and conditioning coaches working in elite-level women’s football?

8. How do you know the club approach is working?

9. Tell me your views on the gathering of all the injury surveillance data?

10. Is there anything else that we haven’t discussed about player availability that you think is important?

**Medical personnel and head coaches interview guide**

1. How do you know players are ready to perform in a game?

2. How do you know players are not ready to perform in a game?

3. (a) What information informs these decisions?

(b) What do you use to measure match performance?

(c) What are your key performance indicators?

4. Who decides if players are available to play?

5. (a) What strategies are used to ensure players are available to play?

(b) Who or what influences these strategies?

6. What are the core competencies required of medical personnel working in elite-level women’s football?

7. What are the core competencies required of strength and conditioning coaches working in elite-level women’s football?

8. How do you know the club approach is working?

9. Tell me your views on the gathering of all the injury surveillance data?

10. Is there anything else that we haven’t discussed about player availability that you think is important?
