# Supplementary material for: Players’, Head Coaches', And Medical Personnels' Knowledge, Understandings and Perceptions of Injuries and Injury Prevention in Elite-Level Women’s Football in Ireland
Source: Sports Med Open. 2023 Jul 29;9:64. doi: 10.1186/s40798-023-00603-6 (PMC10387024; doi:10.1186/s40798-023-00603-6)
Supplement: Supplementary file 3 — Additional file 3. Example Quotes supporting the Theme “Injuries” and its associated Sub-themes. All quotes are categorised according to the three domains of:knowledge;attitudes;practices. C = Head Coaches; M = Medical Personnel; P = Players [file 40798_2023_603_MOESM3_ESM.docx]

**Manuscript Title:** Players’, head coaches and medical personnel knowledge, understandings, and perceptions of injuries and injury prevention in elite-level women’s football in Ireland.

**Journal:** Sports Medicine Open

**Authors:** Dan Horan,^1,5^ Seamus Kelly,^1^ Martin Hägglund,^2,3^ Catherine Blake,^1^ Mark Roe,^1^ Eamonn Delahunt.^1,4^

**Authors’ Affiliations:**

^1^ School of Public Health, Physiotherapy and Sports Science, University College Dublin, Dublin, Ireland

^2^ Football Research Group, Linköping University, Linköping, Sweden

^3^ Division of Physiotherapy, Department of Health, Medicine and Caring Sciences, Linköping University, Linköping, Sweden

^4^ Institute for Sport and Health, University College Dublin, Dublin, Ireland

^5^ Department of Sport, Leisure & Childhood Studies, Munster Technological University, Cork, Ireland

**Corresponding Author Email Address**

Dan Horan: danhoran10@gmail.com

| **Interview** | **Supporting Evidence** |
| --- | --- |
|  |  |
| C3 | I know last year I think when I first spoke to you in our club alone coming into the start of last season, we had three ACL injuries. Touch wood, we haven’t had any the season just gone which is great but to have 3 ACL injuries, two from the 17’s and one from the first team, it is quite a lot you know. |
|  |  |
| C6 | Especially with women’s football I don’t know the reason for it but there is a massive difference between the number of ACL injuries in men’s sport and women’s sport and it’s obviously something to do with the physiology of the women’s body but I think for that reason alone I think girls really need to be clued in as to how to protect themselves from this injury because it is prevalent in the game more so than in the men’s game. |
|  |  |
| M2 | So, the male teams that I had worked with before, you might get … you might not get an ACL at all any season. In my two years working there we had 2 ACL’s and 2 knees as well, 2 meniscal injuries and an MCL. I mean there is a whole host of factors that come into all that and there is loads of research to show that women are more susceptible to ACL stuff for very different reasons |
|  |  |

| **Interview** | **Supporting Evidence** |
| --- | --- |
|  |  |
| C4 | the importance of the game, it can do with certain players and the type of injuries that they have. I am sure a lot of managers are probably guilty of it whether guilty is the right word I don’t know. Sometimes I think you can just get away with it, as I said, with the ankle strain, some of them have gone out and played and they have been fine and they would be fine then the following week. Sometimes they have gone out and it has made them worse. |
|  |  |
| M6 | I just think they are really more highly emotionally strung. When it comes to injuries. Maybe because growing up you know, I know it’s stereotypical but the girlie girl, it’s like oh go away there’s a bee or there’s a wasp and run away from it screaming. Some of them girls might be just good athletes, but they might still be that girlie girl inside. |
|  |  |
| P15 | but if you done the ligaments in your ankle which I have, they’re (the coaches) like sure just strap it up and you can play. |
|  |  |

| **Interview** | **Supporting Evidence** |
| --- | --- |
|  |  |
| C6 | We had one serious ACL and it was just an innocuous injury. She went up for a header and she came down and landed awkwardly. In fairness to him, he has taken that on this year and he said we can try and prevent that so now he is teaching girls how to jump and how to land properly so when he is doing his physio side of it with the strength and conditioning coaches, he will be teaching them how to jump, how to land without their knee buckling so he is taking the fact that we had a girl injured last, he has taken that on this year to try and improve that side of it as well and to try and eradicate that injury. So, he will be proactive that way. |
|  |  |
| M5 | I’m very proactive at the moment with ACL’s so what I’m trying to do is when people are injured whether it be a calf strain or be an ankle injury or shoulder injury, whatever it may be, teach them landing mechanics, more selective muscle activation things and those types of things so that we can try to prevent … I know we can’t bullet proof people for ACLs, but we can definitely prevent them. |
|  |  |
| P13 | Last year in a few games I felt like almost a little twinge in my hamstring but never bad enough that I couldn’t play on it so I would put it to the back of my mind and it would go away on its own then. There’s other ones obviously. I done the ligaments in my ankle and I just couldn’t play on it, physically couldn’t play on it. It’s more whether I physically can or physically can’t play on it. |

| **Interview** | **Supporting Evidence** |
| --- | --- |
|  |  |
| M6 | So basically, what I always factor in when someone is returning, the fear of re-injury, so the holding back. |
|  |  |

| **Interview** | **Supporting Evidence** |
| --- | --- |
|  |  |
| C1 | What I would also be wary of as well, I would be looking at girls and some of them you could see them limping and they won’t pull out because they don’t want to first of all let the team down and they don’t want to let the coaches down |
|  |  |
| C1 | From a physical point of view as well you’ll have some girls, especially the younger girls who want to play through pain and who are injured and you know by looking at them that there could be something wrong but they are a different, they just don’t want to say they are injured and they will play through it |
|  |  |
| C4 | I have found with some women players and it’s the same with men I suppose, I don’t think I can separate it, it is individuals that can give it a bit of a hypochondriac kind of syndrome with them. They feel they’re not right, but they are o.k. I can’t explain this really, it is just observing these individuals. |
|  |  |
| P10 | I think in Ireland there’s a don’t be soft mentality. You’ll be grand, get on with it type of thing |
|  |  |
| P17 | Sometimes players feel that going to the physio is a sign of weakness and that it shows that there’s something wrong with them and they mightn’t be in contention to be selected for the game at the weekend because they have gone to the physio during the week |
|  |  |

| **Interview** | **Supporting Evidence** |
| --- | --- |
|  |  |
| P14 | I know I had an injury the first season and I played through it, but I was o.k. to do that and I feel like I am at a different stage as well. I’m not preparing for international stuff or whatever so I’m o.k. to put my body on the line or if I feel my team needed me to play. You know it’s wrong, the manager knows it’s wrong but sometimes you just, I mean players will fight through pain and that’s just the way it is. |
|  |  |
| P15 | sometimes you play when you really shouldn’t. |
|  |  |

| **Interview** | **Supporting Evidence** |
| --- | --- |
|  |  |
| C2 | A lot of our players play college football as well. There’s a good chunk of our players that play a second sport to a high level as well so at different times in the season you’ve to balance that out so there’s a whole lot of things that come in to play when knowing whether your player is ready to play or not |
|  |  |
| C2 | the light load they were taking in the gym wasn’t preparing them enough for the actions they’d be doing on the pitch. So, we were getting players going to the gym, they were doing good technique but they weren’t going enough load to prepare them for the actions they were going to do on the pitch. |
|  |  |
| M3 | they are constantly working on quad and hamstring… a good ratio between quad and hamstring strength and be sure then that if they do note any deficit that we are picking up on it. Last season I sent one girl in for a Biodex just to be sure because I thought there was a bit of a deficit. |
|  |  |
| P10 | don’t think it’s taken as seriously as it should be. Actually it’s funny because I was going through a menstrual cycle when I got injured and I always remember hearing that knee injuries and bad injuries can happen when you are on it and that’s what happened me and I think it’s something that I definitely look into a bit more because a lot of the girls… I remember when I was at Sunderland three of the girls done their ACL and all three were going through it when it happened, so it is definitely something that needs to be taken more seriously. |
|  |  |
| P16 | I just think a lot of women in the national league probably aren’t strong enough so that’s why they are getting ACL injuries |
|  |  |
| P17 | Obviously at different stages of the cycle you could be more susceptible to injuries but also in terms of performance as well. |
|  |  |

| **Interview** | **Supporting Evidence** |
| --- | --- |
|  |  |
| C1 | during the session, if I am honest with you, during the session, because I am the type of coach who likes intensity and all that, I probably push the girls a little bit too hard. I would be a small bit old school that way and I am always on the edge in relation to maybe someone could get injured |
|  |  |
| C4 | Certain players are more vulnerable than others. Again, that can all be connected to the menstrual cycle as well. It would be good to know what players when they do get injured was it that time of the month when they got injured. It would be good to have information around that. |
|  |  |
| C5 | I think if you overdo it on astro-turf and you do your two hours, the legs can get very tired. I am mindful that I would never do more than an hour and a half probably slightly less than that and I think they feel better after doing that |
|  |  |
| M2 | Obviously without having your GPS stuff you can’t see the science side of it but from my point of view, it would seem like that the players weren’t adapting to a new stimulus and some of them just couldn’t deal with it because they just didn’t have the general capacity and robustness to be able to deal with the stress you are placing on them. |
|  |  |
| P11 | Like I feel like some players, some players are actually doing too much and cause injury for themselves, like unnecessary injury. |
|  |  |
| P12 | I don’t think people are educated on it, I don’t think they have an idea about it and yeah I think it’s the education of it, I just don’t think it’s up to that standard really. |
|  |  |
| P8 | I have always found that if I am on my period I will play quite well, I don’t know what it is. Your senses are heightened or whatever I think it is… once you have your Panadol, I think you’re fine. You’re good to go.  I think it was because I read on a packet of pads sometime when I was younger that it heightens your alertness and ever since then I have believed that I play better so again it is a mental thing. Whereas if someone thinks oh I’m bloated, I’m suffering, I’m not going to play well. |
|  |  |

| **Interview** | **Supporting Evidence** |
| --- | --- |
|  |  |
| M1 | You have to be able to condition players to last the length of the game. With 90 minutes games it is a pretty prolonged activity but also I mean if your player isn’t fit or potentially at risk of picking up injuries towards the ends of games, typically hamstring injuries if they are coming towards the ends of games. If we are just trying to build up that robustness and that capacity that we are trying to protect players from injury really. That goes from the strength and conditioning stuff as well as general fitness levels. |
|  |  |
| M2 | if you have a grade II hamstring and they break down again in three month’s time with another hamstring you have got to look at, there’s some factors here that I have missed, whether it be in terms of load management or are they strong enough, are they robust enough. You have got to look on that side of things. You are constantly re-evaluating yourself and the principals I would have had when I started working with the women’s team were very different to what I had towards the end. |
|  |  |
| M3 | I think the only thing that they would be involved in would be college football or college sports. Some of them are on scholarships so I would liaise with some of the college managers, they have been quite good. They would send me on their fixtures just to see how they are tying in with our training sessions or our fixtures and if we feel that there is too much of a demand, we obviously want them to step back from the college or only play say 20 minutes of a college match if we know they are coming into a training session then the following day. So, we would liaise with the college managers with things like that. |
|  |  |
| M3 | If I know that the player is on scholarship I would say to them ok, would the coach be open to me contacting them about your training schedule and tying it in with our training schedule and then if you do come into any injuries, is there someone that I can contact on a medical side or a medical team, that side so that we’re all working off the same concept or ethos about rehabilitation on a particular injury. |
|  |  |
| P10 | I have kind of been a little bit stupid and naïve in terms of just trying to get through injuries whereas now if I feel a niggle, I’ll either not do a session or I’ll try and do something that isn’t going to put a load on the area that’s injured, if that make sense. |
|  |  |
| P10 | I think it is something that is slowly coming in to the game. I think XXXX is definitely very for it… whenever we come into camp we have to fill in a questionnaire about when we have had it last and when we’re due and the first day of it we’re not allowed train, we’re given a lighter session to do so it would be something that she definitely takes serious but I don’t know whether it is a taboo thing that people don’t want to talk about it. |
|  |  |
| P3 | the manager would know our schedules. So, say if we are playing in college and if we had a game with XXXX at the weekend and then a game in college on the Wednesday or whatever we might take off the Thursday of that week |
|  |  |
