# Supplementary material for: Players’, Head Coaches', And Medical Personnels' Knowledge, Understandings and Perceptions of Injuries and Injury Prevention in Elite-Level Women’s Football in Ireland
Source: Sports Med Open. 2023 Jul 29;9:64. doi: 10.1186/s40798-023-00603-6 (PMC10387024; doi:10.1186/s40798-023-00603-6)
Supplement: Supplementary file 4 — Additional file 4. Example Quotes supporting the Theme “Prevention of Injuries” and its associated Sub-themes. All quotes are categorised according to the three domains of:knowledge;attitudes;practices. C = Head Coaches; M = Medical Personnel; P = Players [file 40798_2023_603_MOESM4_ESM.docx]

**Manuscript Title:** Players’, head coaches and medical personnel knowledge, understandings, and perceptions of injuries and injury prevention in elite-level women’s football in Ireland.

**Journal:** Sports Medicine Open

**Authors:** Dan Horan,^1,5^ Seamus Kelly,^1^ Martin Hägglund,^2,3^ Catherine Blake,^1^ Mark Roe,^1^ Eamonn Delahunt.^1,4^

**Authors’ Affiliations:**

^1^ School of Public Health, Physiotherapy and Sports Science, University College Dublin, Dublin, Ireland

^2^ Football Research Group, Linköping University, Linköping, Sweden

^3^ Division of Physiotherapy, Department of Health, Medicine and Caring Sciences, Linköping University, Linköping, Sweden

^4^ Institute for Sport and Health, University College Dublin, Dublin, Ireland

^5^ Department of Sport, Leisure & Childhood Studies, Munster Technological University, Cork, Ireland

**Corresponding Author Email Address**

Dan Horan: danhoran10@gmail.com

| **Interview** | **Supporting Evidence** |
| --- | --- |
|  |  |
| C3 | I think there would be a big indicator there that if someone’s fitness test results aren’t, if they are less than what they were previously six weeks ago then you know there is something not right there. Whether she has picked up a niggle or whether there is something personal going on as well. |
|  |  |
| M5 | Well in terms of energy levels for sure. Monitoring stress levels with the female athletes probably more so because of just having slightly higher risks of low bone mass, the female athlete triad is something I have in mind when I’m working with female players. Especially some of the higher-level ones. Looking at their weight. |
|  |  |
|  |  |
|  |  |

| **Interview** | **Supporting Evidence** |
| --- | --- |
|  |  |
| M1 | I would like to have a system in place. Say their RPE, their training load for that week where you can see constant feedback coming into you because it seems to be always the same sort of chat up when the player comes in for an injury. You ask what’s the problem, tight calves, what were you doing this week? And then they go into a rant about I played basketball Monday, Gaelic on Tuesday. So, I think it is something that should be looked at. |
|  |  |
| M4 | We were trying to monitor it (training load), yes but I felt it was falling on deaf ears at times. |
|  |  |
| P10 | if it’s the same injuries re-occurring it’s obviously something that the coaches are doing wrong or not doing right so I think it is something that needs to be looked at. |
|  |  |

| **Interview** | **Supporting Evidence** |
| --- | --- |
|  |  |
| C2 | One thing we also did was when players put in something about their day. Let’s say when you’re allowed leave a comment. It might be something like I’m tired and drained from college. I was up late studying for an exam the night before. That we tended to then use it as something to communicate with them on. Well done for telling us that. It’s good we know that going in to this week. We’ll be able to manage it. So I suppose a big thing for us was to make them positive on the system rather than why aren’t you filling it in! So we found that worked well. |
|  |  |
| M3 | We do an awful lot of fitness testing and I suppose general testing. So, functional movement screening as well so if I did see a player that was not performing to what I would think to that particular player’s peak or their standard I would probably pull them off and like that if they were lacking power or lacking speed, is it a hamstring issue, their last sit and reach test was this or the last long standing jump was that. |
|  |  |
| M3 | We do the sit and reach test. So, we have the player sitting and we have a box and we measure how far that they can reach so it would put an awful lot of pressure on the hamstrings and things like that and posterior chain mobility, so we do that. We do the standing long jump so setting out the measuring tape and just seeing how far they can just from one position to jump forward. So, again, a good bit of power and plyometric background to that one. We would have the basic fitness testing on the pitch. So, Illinois left, Illinois right, 20 meters, even just a simple 1 kilometre run and just timing them to see how good they are and then some agility testing. |
|  |  |
| M3 | they also have a log in, a player log in where they can report different things like their mood, how they’re feeling, what they ate, what they drank so it is a good variation. A good reliable source where you can look back and think o.k. she didn’t do so well or she didn’t train that day but if you go back on to her log you might see that she was feeling a bit under the weather or there could have been a family issue going on and you can correlate them all together then. |
|  |  |
| M5 | Testing at the start of the year, you can set up a team plan working on I suppose their sports specific needs and then retests throughout the year. So, it’s motivational, there’s competitiveness and then you are also making sure you’re keeping track of … you know who is doing it and who is not. So, that’s really important to know for when injuries do happen to have that information. |
|  |  |
| P1 | So actually trialing this new app now for the next month. So it’s actually a very good app. It records everything like your sleep, your wellness, stress levels, everything and then obviously exactly what training you are doing. Like pitch sessions, recovery, foam rolling, strength, mobility, everything. Like you just fill out exactly what you are doing. And the managers can review the app. |

| **Interview** | **Supporting Evidence** |
| --- | --- |
|  |  |
| C4 | Well it helps in terms of the history of the player, maybe if they are susceptible in getting certain injuries and what their reaction is to it and what’s their timeframe in getting back from it. |
|  |  |

| **Interview** | **Supporting Evidence** |
| --- | --- |
|  |  |
| C7 | it’s good, but it needs to be for a reason. It’s needs to inform something; it needs to inform change. Again, it’s like what would it allow us to do? That’s really where I would like to see the comeback on it. |
|  |  |
| M5 | Gathering data is essential if you want to make changes, you have to know. You start with awareness of what a situation is, what currently is happening before you can make changes so it is the right way to start |
|  |  |
| M7 | Look it is definitely, the more data we have for anything, the better it is, the more we can construct better rehab programmes but it’s just getting the clubs to buy into using it I suppose. |
|  |  |
| P16 | I think going forward to improve our league and to improve our structures, I think this (ongoing injury surveillance) needs to be done because if we leave this go for another few years, we’re going to be back in square one that people are injured, people aren’t happy and that we don’t know why all these injuries are the same injuries that are reoccurring. |

| **Interview** | **Supporting Evidence** |
| --- | --- |
|  |  |
| M2 | I think having that research there so you can take little chunks of it and apply it gradually to your practice I think it is massively important. |
|  |  |
| M8 | Being honest, I think I was the only one that really had an interest obviously.… it was tough. Because obviously their (coaches) priorities are different to mine. And obviously winning games and making the club work on the business side of things. Its different interests and I suppose the science and that kind of getting the data and stuff like that. Maybe it was hard at that point for it to be considered as something that really was of value compared to maybe where else they would want to put their money if they had more. |

| **Interview** | **Supporting Evidence** |
| --- | --- |
|  |  |
| C1 | The FIFA 11 one yes. It would be a breakdown of that now, not a complete, but it would be a breakdown of that, yes. |
|  |  |
| C5 | I like the FIFA 11+ and I think that they should look at that and they should be doing certain exercises and we have given them the videos to make sure that if they are doing a little bit of work by themselves then I quite like it. |
|  |  |
| M2 | I mean there is a whole host of factors that come into all that and there is loads of research to show that women are more susceptible to ACL stuff for very different reasons but I mean, yes o.k. you are going to get them and you try to implement as much, your proprioceptive stuff, your plyometric landing mechanics and try and help in preventing that stuff. |
|  |  |
| M5 | I know a lot of people use FIFA 11+ and things like that for setting up programs as well so obviously I would borrow some stuff from that. |
|  |  |

| **Interview** | **Supporting Evidence** |
| --- | --- |
|  |  |
| C1 | To be honest with you, the big thing for me is the pre-activation exercises at the start of training |
|  |  |
| M7 | I don’t like doing exercises just for the sake of them. There has to be some kind of a reason. Some kind of an injury that you are trying to prevent and some kind of a mindset behind it |
|  |  |
| P10 | if it’s the same injuries re-occurring it’s obviously something that the coaches are doing wrong or not doing right |
|  |  |
| P13 | We just meet up 3 times a week for training and there’s no injury preventions being sent out or anything like that. No conditioning programs. |
|  |  |

| **Interview** | **Supporting Evidence** |
| --- | --- |
|  |  |
| C2 | we start to observe things about their game as they start to pick up injuries and find out their injury history that their programme develops into what’s more individually in tune with them. |
|  |  |
| C3 | In December we brought the girls in for a day before pre-season into the gym here and he went through injury prevention exercises with them. We videoed the exercises with them and we sent them out to every player with a breakdown of why they are doing it and what they are doing. |
|  |  |
| C6 | now he is teaching girls how to jump and how to land properly so when he is doing his physio side of it with the strength and conditioning coaches, he will be teaching them how to jump, how to land without their knee buckling so he is taking the fact that we had a girl injured last, he has taken that on this year to try and improve that side of it as well and to try and eradicate that injury. So, he will be proactive that way. |
|  |  |
| M6 | I’m very proactive at the moment with ACL’s so what I’m trying to do is when people are injured whether it be a calf strain or be an ankle injury or shoulder injury, whatever it may be, teach them landing mechanics, more selective muscle activation things and those types of things so that we can try to prevent … I know we can’t bullet proof people for ACLs, but we can definitely prevent them. |
|  |  |
| M7 | I suppose I would say for instance, a hamstring injury. Yes, they will do the rehab, they will progress on to the eccentric stuff but then I would probably try and get them to do the Nordics for example, an eccentric program for about six weeks. A maintenance program for about six weeks. |
|  |  |
| M7 | I know the research might be a bit inconclusive but still I would try and get them just kind of maintenance rubs, pre-season maybe, get everybody at least one rub a week. I would just do up a list so that will… I find it helps anyway. |
|  |  |
| P8 | We haven’t done any of those injury prevention stuff. I actually don’t think I’ve really ever done it. |
|  |  |
| M4 | I felt it was a very poor warm-up, especially on match days you could see that coming through that they didn’t have a structured warm-up at any stage and I just felt it was a little bit lacking on those sides. Match days particularly, they didn’t even look like they were getting warm before the warm up. It felt very slow, it felt very sluggish. Wasn’t set up as a structured warm up is. The best way I can describe it would be your normal junior B GAA team, right lads c’mon out we go, a few high knees, a few heel kicks, off we go. From there not being a warm up I have noticed a few soft tissue injuries that I feel shouldn’t have happened had they been properly warmed up. |
