# Supplementary material for: Players’, Head Coaches', And Medical Personnels' Knowledge, Understandings and Perceptions of Injuries and Injury Prevention in Elite-Level Women’s Football in Ireland
Source: Sports Med Open. 2023 Jul 29;9:64. doi: 10.1186/s40798-023-00603-6 (PMC10387024; doi:10.1186/s40798-023-00603-6)
Supplement: Supplementary file 5 — Additional file 5. Example Quotes supporting the Theme “Injury Management” and its associated Sub-themes. All quotes are categorised according to the three domains of:knowledge;attitudes;practices. C = Head Coaches; M = Medical Personnel; P = Players [file 40798_2023_603_MOESM5_ESM.docx]

**Manuscript Title:** Players’, head coaches and medical personnel knowledge, understandings, and perceptions of injuries and injury prevention in elite-level women’s football in Ireland.

**Journal:** Sports Medicine Open

**Authors:** Dan Horan,^1,5^ Seamus Kelly,^1^ Martin Hägglund,^2,3^ Catherine Blake,^1^ Mark Roe,^1^ Eamonn Delahunt.^1,4^

**Authors’ Affiliations:**

^1^ School of Public Health, Physiotherapy and Sports Science, University College Dublin, Dublin, Ireland

^2^ Football Research Group, Linköping University, Linköping, Sweden

^3^ Division of Physiotherapy, Department of Health, Medicine and Caring Sciences, Linköping University, Linköping, Sweden

^4^ Institute for Sport and Health, University College Dublin, Dublin, Ireland

^5^ Department of Sport, Leisure & Childhood Studies, Munster Technological University, Cork, Ireland

**Corresponding Author Email Address**

Dan Horan: danhoran10@gmail.com

| **Interview** | **Supporting Evidence** |
| --- | --- |
|  |  |
| C6 | They have to obviously be fully qualified |
|  |  |
| P1 | I think it should be a qualified physio. |
|  |  |
| P3 | we’re the elite level in this whole thing why shouldn’t we have a fully trained physio. I don’t see why there shouldn’t be, I really don’t. |
| P5 | Well to be a chartered physio is obviously number one. You can have some ones maybe doing an internship or a similar degree or something like that but I think you have to be a chartered physio |
|  |  |

| **Interview** | **Supporting Evidence** |
| --- | --- |
|  |  |
| C6 | They have to obviously be fully qualified |
|  |  |
| M1 | So, I am coming in as a student saying that this player can’t play. He can obviously say oh you don’t know what you’re saying, I know these players, she’s well able, even though I know full well she’s injured, she can’t play so that could easily cause conflict. |
|  |  |
| P1 | I think it should be a qualified physio. |
|  |  |
| P14 | I don’t know if he has his qualification, I’m not aware, I wouldn’t say that he is fully qualified. |
|  |  |
| P3 | We have had a few who have come, and I don’t know, they are not used to working with women or girls or what. They are male physios now I’m going to say because the female ones would be better at this, but it feels like they are treating you like you’re stupid like and I don’t think they mean to do that but they say things very simplistic like or sometimes it is like they are talking slower to you or something. You are kind of going look I’m not thick like. |
|  |  |

| **Interview** | **Supporting Evidence** |
| --- | --- |
|  |  |
| C1 | At the moment, they are students. Because we don’t have an onsite fully qualified physiotherapist |
|  |  |
| M1 | So I am normally with one other student for the duration of the training so there’s always going to be one of us pitch side, if you know what I mean so if someone say does get a serious injury there would be one in the clinic room, one pitch side, we never neglect the pitch side if you know what I mean. |
|  |  |
| M1 | So, our supervisor wouldn’t actually be there at the moment. … is his name; we communicate with him through phone or via email. We would have to update how many hours we did, what injuries we saw. That’s weekly, we just send him individual messages. |
|  |  |
| P1 | I feel like they have seen an opportunity that a student physio is available and just oh that will save us money, do you know what I mean? |
|  |  |
| P12 | I have actually no idea. I don’t know if it’s funds, I don’t know if it’s, well in our particular case … does both teams, so that's probably not ideal. Do you know, frustratingly, more than likely the men come first. That’s just very frustrating. But my fear is, if anything happened to one of us, of a training night you know yourself, a clash of heads, bad landing and someone who I suppose has the highest qualifications, should really be there. And this is something we have mentioned for years trust me. |
|  |  |
| P13 | He’s not there every night but we have actually … we have a link with … at the moment, so we have had student physios there every night so there has been a physio there every night but the club physio has been there maybe once a week. |
|  |  |
| P15 | Last year our… physio is not the right word because it’s not what either of them were, that’s not their … they might have been physical therapists or something, not chartered physio but last year the guy that was doing it was not even close to being anybody that you would trust to decide whether you should be playing or not playing. |
|  |  |
| P3 | We had a lot of physios who are trainee physios as well. Don’t get me wrong, everyone has to learn, you have to go on placement which is fine but sometimes they are given sole control for our team for a match and that’s not on. |
| P3 | They are not fully trained, if something happens and for instance they haven’t covered it or they haven’t come across it before how can they number one treat it and number two assess whether you can play on or not. |

| **Interview** | **Supporting Evidence** |
| --- | --- |
|  |  |
| P11 | I think the physio should be there at every session. |
|  |  |
| P12 | What if one of the players fell to the floor with their chest or anything like, that’s when you kind of think like Jesus like … We should really have a physio every session like. Seasons I have been involved in there wasn’t physios at sessions at all like. |
|  |  |
| P5 | I think it is 100% required. If it is to be maintaining a professional standard for women’s soccer, it is an integral part of it really, I don’t think it would be safe to be doing high intensity training and competitive matches without physios on hand to use their expertise and to help out. |
|  |  |

| **Interview** | **Supporting Evidence** |
| --- | --- |
|  |  |
| C4 | It shows that if they do get an injury or they have a strain that there’s someone there to look after them. It shows that we care about them, it shows there’s some resources being put in to look after them. I think there’s a big part of that and that’s why it is important to the players. |
|  |  |
| C4 | Some players like to go to a physio, like to have a physio there so I would expect the physio there early before the … half an hour anyway, 45 minutes before the session starts so players can turn up early if they need him. Even if they need to go in to get that magic rub, if it is as something as simple as that, to me that’s good, that’s ticking a box. |
|  |  |

| **Interview** | **Supporting Evidence** |
| --- | --- |
|  |  |
| M1 | So I am normally with one other student for the duration of the training so there’s always going to be one of us pitch side, if you know what I mean so if someone say does get a serious injury there would be one in the clinic room, one pitch side, we never neglect the pitch side if you know what I mean. |
|  |  |
| M4 | I was only in for the match day kind of trauma covering and one evening a week with them training. They trained two other nights. |
|  |  |
| P12 | I have actually no idea. I don’t know if it’s funds, I don’t know if it’s, well in our particular case … does both teams, so that's probably not ideal. Do you know, frustratingly, more than likely the men come first. That’s just very frustrating. But my fear is, if anything happened to one of us, of a training night you know yourself, a clash of heads, bad landing and someone who I suppose has the highest qualifications, should really be there. And this is something we have mentioned for years trust me. |
|  |  |
| P13 | He’s not there every night but we have actually … we have a link with … at the moment, so we have had student physios there every night so there has been a physio there every night but the club physio has been there maybe once a week. |
|  |  |
| P16 | So, there is one physio for I’d say about 5 teams. |
|  |  |
| P3 | We have a physio at home games, they are always at the home games, but we don’t have our own physio at away games. |
|  |  |
| M6 | The vital 48 hours is what is missing 80% of the time. The cooling down period, that 48 hour period that you can start getting early rehabilitation… just even an isometric hold into a muscle which will stimulate the nervous system and might take five days off the recovery time, it just doesn’t happen |
|  |  |
| P4 | I suppose last year there was one physio for the seniors and they were also looking after all of the underage teams, so the boys and the girls. So, it was kind of… they were completely overrun so it was hard to get proper time to really look into it (injury) |
|  |  |
| P4 | Instead of having maybe two or three to keep an eye on or look after, there was the 13’s, the 15’s, the 17’s, the 19’s and the girls seniors, probably too much…. just people weren’t getting individual time to go through rehab programs or exercises. I suppose obviously myself, my own injury I suppose would probably have… I wasn’t able at the time to really dig into and find out what is causing it |
|  |  |
| M6 | What I would have loved with our girls, it’s just too time consuming for one person to do… a baseline SCAT 5 pre-season with the pre-season screening. It would have been fantastic to have them baseline measures that if you can come back to if someone is suspected of a concussion that you can compare. |

| **Interview** | **Supporting Evidence** |
| --- | --- |
|  |  |
| C6 | They have to obviously be fully qualified |
|  |  |
| P1 | I think it should be a qualified physio. |
|  |  |
| P3 | They are not fully trained, if something happens and for instance they haven’t covered it or they haven’t come across it before how can they number one treat it and number two assess whether you can play on or not. |
|  |  |
| P3 | we’re the elite level in this whole thing why shouldn’t we have a fully trained physio. I don’t see why there shouldn’t be, I really don’t. |
|  |  |
| P5 | Well to be a chartered physio is obviously number one. You can have some ones maybe doing an internship or a similar degree or something like that but I think you have to be a chartered physio |
|  |  |
| M6 | The next thing then is to understand concussion. Anything else I think anyone can wait. You need to be upping your game with those treatments of concussion as well and you need to have an in-depth SCAT 5 now. |

| **Interview** | **Supporting Evidence** |
| --- | --- |
|  |  |
| P1 | I feel like they have seen an opportunity that a student physio is available and just oh that will save us money, do you know what I mean? |
|  |  |
| P14 | I don’t know if he has his qualification, I’m not aware, I wouldn’t say that he is fully qualified. I just don’t think he is. |
|  |  |
| P3 | We have had a few who have come, and I don’t know, they are not used to working with women or girls or what. They are male physios now I’m going to say because the female ones would be better at this, but it feels like they are treating you like you’re stupid like and I don’t think they mean to do that but they say things very simplistic like or sometimes it is like they are talking slower to you or something. You are kind of going look I’m not thick like. |
|  |  |
| P3 | I don’t think it is fair on them either because we are obviously frustrated that they are not qualified…obviously they are going to be cautious because they don’t know exactly what it (the injury) is and I can’t blame them for being cautious but they shouldn’t be put in to that position either like |
|  |  |
| C6 | first of all be proactive and keep the girls on the pitch as much as possible and have things in place to reduce the risk of injuries but also I want somebody there who will give it to me straight and not be telling me what I want to hear and have the best interest. |

| **Interview** | **Supporting Evidence** |
| --- | --- |
|  |  |
| C1 | At the moment, they are students. Because we don’t have an onsite fully qualified physiotherapist. |
|  |  |
| M1 | So I am normally with one other student for the duration of the training so there’s always going to be one of us pitch side, if you know what I mean so if someone say does get a serious injury there would be one in the clinic room, one pitch side, we never neglect the pitch side if you know what I mean. |
|  |  |
| P12 | Do you know, frustratingly, more than likely the men come first. That’s just very frustrating. But my fear is, if anything happened to one of us, of a training night you know yourself, a clash of heads, bad landing and someone who I suppose has the highest qualifications, should really be there. And this is something we have mentioned for years trust me. |
|  |  |
| P13 | so we have had student physios there every night so there has been a physio there every night but the club physio has been there maybe once a week. |
|  |  |
| P15 | Last year our… physio is not the right word because it’s not what either of them were, that’s not their… they might have been physical therapists or something, not chartered physio but last year the guy that was doing it was not even close to being anybody that you would trust to decide whether you should be playing or not playing. |
|  |  |
| P15 | So, like you get a bang or an elbow and maybe you’re not quite right afterwards. So, I suppose light concussion… again, if you were in our situation last year and that physio certainly wouldn’t know how to deal with that properly and take the person out |

| **Interview** | **Supporting Evidence** |
| --- | --- |
|  |  |
| M1 | the players health is their wellbeing |
|  |  |
| M1 | it is our priority to protect players |
|  |  |
| M5 | I think player welfare is number one, so I am very happy to stand up for player if I feel they are a high risk of injury. |
|  |  |
| M5 | Player welfare. Look after the players health. I suppose as a physiotherapist, I have trained. I didn’t train medically but I trained in a medical school and our approach was very much patient welfare. My code is looking after the player and making sure that they are safe |
|  |  |
| P16 | I think the player welfare should be the big one in that if the player isn’t right to play, the player shouldn’t play. |
|  |  |

| **Interview** | **Supporting Evidence** |
| --- | --- |
|  |  |
| C4 | Sometimes with players who I don’t want to pick if he says there’s a strain or even during training, I will say right well probably let them know that it’s a little bit worse than it is maybe. It gives me a way out of not picking them then because they’re not right. I could do that, whether it is right or wrong. Then the opposite if I feel the player and the player wants to play and the physio says it’s not too bad, I would take a chance with them. Depending on how badly I need that player to play. |
|  |  |
| C4 | It’s tricky. I would be nearly saying to the players here tell him you have a little strain and you can’t train in the college you know. |
|  |  |
| C5 | So, I think you have got to be able to say that I trust you and you are in charge of that and whatever you say is important to me. So, whilst I will ask the questions, I won’t interfere with something that I don’t really understand as well as probably the other person does. |
|  |  |
| M1 | So, I am coming in as a student saying that this player can’t play. He can obviously say oh you don’t know what you’re saying, I know these players, she’s well able, even though I know full well she’s injured, she can’t play so that could easily cause conflict. |
|  |  |
| M1 | Being confident in treatment of injuries, being confident in diagnosis as well because even if you are unsure say of the differentials, even by just acting, being cool with them and sounding confident it will give player reassurance and faith |
|  |  |
| M3 | I came in after a male physio and again no disrespect to the male physio but I found that an awful lot of the girls came to speak to me because they couldn’t maybe speak to him in the past and I found that I had a good few players chit chat to me about day to day things and it wouldn’t be affecting their physical play but maybe mentally they might just want to have a chat and then just run on to the pitch and know that they have a clear head for it. |
|  |  |
| M5 | The management would have been very good, they would have always listened and taken on board what I said but again the final decision is always with them and the player. I felt my role was more as advice or as I don’t know if I had as much input to the team or as a physio does with some professional teams. |
|  |  |
| M5 | in an ideal world, if you can actually have a meeting between all specific people. So, have the player, the manager and myself there. Talk about this is what has happened, this is the injury assessment that we have found, this is the injury, this is where we need to get to, this is probably the timeframe. |
|  |  |
| M5 | The fact that you are having the conversation between all three would be nice because then there is no communication going on between the player and the manager separate from the medical team I think it’s just transparency, it’s so important and the player knows where they stand and they feel safer. |
|  |  |
| M5 | if I didn’t have clear communication between me and the management team, yes then all of the conversations around player welfare, around training load, around injury rehab and return to play, return to training, all of those conversations lose value. |
|  |  |
| M7 | You have to be a bit of fun; you have to be a bit of craic. You have to look after the psychological side of the injury as well but at the same time. I always tell the player, you know your body better than me but I know how your body heals better than you do. |
|  |  |
| P13 | I would just keep it to myself unless it gets bad and I think it needs to be looked at. I just feel like it’s not worth the attention of the physio. I know myself, if it’s bad I’ll go to him, but I’ll know if I can play on it, I can keep it to myself. |
|  |  |
| P16 | The manager would ask if we strap it would you play because we really need you for the game whereas I think that responsibility should be on the physio. Obviously getting treated by the physio and him telling you no … you are definitely not playing. Whereas it is kind of up in the air really. |
|  |  |

| **Interview** | **Supporting Evidence** |
| --- | --- |
|  |  |
| C1 | If a physio or a physical therapist says to me look this player can’t play, I would never force that player to play. |
|  |  |
| C2 | If our physio says our player is unavailable, our player is unavailable. |
|  |  |
| C5 | I will always speak to them during the week and if he says they’re not going to be fit, they’re not going to be fit. |
|  |  |
| C5 | I will not put a player on the pitch unless he says she’s fine to play. |
|  |  |
| C6 | we don’t put pressure on them to play all the time and we’re very, very clear of the fact that if they are injured they need to step out. |
|  |  |
| C6 | If the physio comes to me and say she can’t play, then she can’t play. If the physio says its touch and go then she doesn’t play. That’s the way I work. Unless she has a full clean bill of health, she doesn’t play. |
|  |  |
| P16 | You are kind of pressured. Obviously, you want to play but you are kind of being told in the nicest way possible ‘oh you do know your position is up for grabs’. We have five decent defenders if you are not playing then obviously you are going to be the one that’s gone. |
|  |  |
| P16 | I would say very badly. You go on to the physio table and you are injured. You are not told yourself what is actually what’s wrong with you. You are being told, yes it’s a bit tight or you are not training and then he will turn around and just say you get yourself ready to go out and not train and you meet the manager on the way in and he’s like are you not training and you go no and then he’ll go in and speak to the physio so it’s not really like, the three of you don’t go sit down and talk about it. |
|  |  |
| P3 | The manager will consult with our physio if someone is carrying a knock, they would talk to the physio, they would talk to the players themselves but I suppose ultimately it is the managers decision but they would have a lot of input from the physio in fairness. |
|  |  |
| P4 | It is the physio’s call really and if they feel you are not fit enough to play then I think everyone respects that decision and that call. It is made for your best interest and I think everyone kind of knows that. |
|  |  |
| C7 | It’s the old fashioned one isn’t it. They all tell the physio everything and the physio tells you everything. Ha ha ha. Every player knows it as well. You don’t want to go up to the manager and say you can’t take part in a certain part of the session, so you get the physio to do it. |
|  |  |
| C7 | for example if they are telling me it’s just a little bit tight after training when they stretch the next day. When they are with the physio then they’re saying ‘oh it’s killing me for a few days after’. The physio gets the full story. It’s great if you have got a physio that is getting that information from players and the players trust. So, it’s all about relationships and then you’re getting that information back. The players know you get it back, but they also know then that you address it with the player in a way where you don’t come up to them and say Jesus I know you’re really struggling. You address it in terms of their workload, and you adapt it to suit them and they know it’s been addressed |
