# Supplementary material for: Players’, Head Coaches', And Medical Personnels' Knowledge, Understandings and Perceptions of Injuries and Injury Prevention in Elite-Level Women’s Football in Ireland
Source: Sports Med Open. 2023 Jul 29;9:64. doi: 10.1186/s40798-023-00603-6 (PMC10387024; doi:10.1186/s40798-023-00603-6)
Supplement: Supplementary file 6 — Additional file 6. NVIVO coding [file 40798_2023_603_MOESM6_ESM.docx]

**Manuscript Title:** Players’, head coaches and medical personnel knowledge, understandings, and perceptions of injuries and injury prevention in elite-level women’s football in Ireland.

**Journal:** Sports Medicine Open

**Authors:** Dan Horan,^1,5^ Seamus Kelly,^1^ Martin Hägglund,^2,3^ Catherine Blake,^1^ Mark Roe,^1^ Eamonn Delahunt.^1,4^

**Authors’ Affiliations:**

^1^ School of Public Health, Physiotherapy and Sports Science, University College Dublin, Dublin, Ireland

^2^ Football Research Group, Linköping University, Linköping, Sweden

^3^ Division of Physiotherapy, Department of Health, Medicine and Caring Sciences, Linköping University, Linköping, Sweden

^4^ Institute for Sport and Health, University College Dublin, Dublin, Ireland

^5^ Department of Sport, Leisure & Childhood Studies, Munster Technological University, Cork, Ireland

**Corresponding Author Email Address**

Dan Horan: danhoran10@gmail.com

| **Theme** | **Sub-theme** | **Lower order theme** | **Number of participants** | **Number of quotes** |
| --- | --- | --- | --- | --- |
| Injuries |  |  |  |  |
|  | Common injuries |  | 19 (4C^a^; 5M^b^; 10P^c^) | 77 |
|  |  | ACL tear  Lateral ankle ligament sprain  Hamstring tear  Quadriceps tear  Adductor tear  Concussion  Knee meniscus & cartilage injuries  Haematoma  MCL tear  Fracture  Bone stress  Calf tear | 15 (3C; 3M; 9P)  9 (1C; 2M; 6P)  9 (2C; 3M; 4P)  4 (1C; 2M; 2P)  3 (1M; 2P)  3 (2M; 1P)  2 (2M)  2 (1M; 1P)  2 (1M; 1P)  1 (1P)  1 (1M)  1 (1P) | 25  12  17  5  5  4  2  2  2  1  1  1 |

^a^Head Coaches; ^b^Medical personnel; ^c^Players

| **Theme** | **Sub-theme** | **Lower order theme** | **Number of participants** | **Number of quotes** |
| --- | --- | --- | --- | --- |
| Injuries |  |  |  |  |
|  | Player availability |  | 18 (4C^a^; 1M^b^; 13P^c^) | 38 |
|  |  | Availability for matches  Availability for training | 16 (3C; 1M; 12P)  6 (4C; 2P) | 26  12 |

^a^Head Coaches; ^b^Medical personnel; ^c^Players

| **Theme** | **Sub-theme** | **Lower order theme** | **Number of participants** | **Number of quotes** |
| --- | --- | --- | --- | --- |
| Injuries |  |  |  |  |
|  | Injury risk factors |  | 28 (4C^a^; 8M^b^; 16P^c^) | 162 |
|  |  | Menstrual cycle beliefs  Conflict with college football  Poor athletic development of females  WNL players playing multiple sports  Challenge of discussing menstrual cycle  Training load management  Previous injury  Fitness level of players  Sprinting and high speed running  Fixture congestion  Change of manager  Joint laxity  Hamstring quadriceps ratio  Muscle tightness  Playing surface | 16 (2C; 4M; 10P)  15 (2C; 3M; 10P)  7 (3M; 4P)  7 (1C; 2M; 4P)  6 (1C; 5P)  6 (1M; 5P)  4 (1C; 2M; 1P)  3 (1M; 2P)  3 (3P)  2 (2P)  1 (1M)  1 (1M)  1 (1M)  1 (1P)  1 (1C) | 37  39  14  12  12  21  5  5  6  2  4  1  2  1  1 |

^a^Head Coaches; ^b^Medical personnel; ^c^Players

| **Theme** | **Sub-theme** | **Lower order theme** | **Number of participants** | **Number of quotes** |
| --- | --- | --- | --- | --- |
| Prevention of injuries |  |  |  |  |
|  | Monitoring players |  | 20 (2C^a^; 6M^b^; 12P^c^) | 46 |
|  |  | Training load monitoring  Wellness monitoring  Fitness testing  RPE  Musculoskeletal screening | 12 (1C; 4M; 7P)  8 (1C; 3M; 4P)  5 (1C; 2M; 2P)  4 (4P)  2 (2M) | 14  14  8  7  3 |

^a^Head Coaches; ^b^Medical personnel; ^c^Players

| **Theme** | **Sub-theme** | **Lower order theme** | **Number of participants** | **Number of quotes** |
| --- | --- | --- | --- | --- |
| Prevention of injuries |  |  |  |  |
|  | Injury surveillance |  | 18 (2C^a^; 4M^b^; 12P^c^) | 33 |
|  |  | Injury prevention guidance  Injury risk factors  Re-injuries  Evidence to support funding requests  ACL injury  Hamstring injury  Ankle injury  Comparison to other leagues  Rehabilitation  Player to player education  Club staff influence on injury incidence  Value of injury surveillance | 11 (1C; 2M; 8P)  3 (1C; 2P)  3 (1M; 2P)  3 (1M; 2P)  2 (1M; 1P)  2 (2M)  2 (1M; 1P)  1 (1C)  1 (1M)  1 (1P)  1 (1P)  1 (1M) | 13  3  3  3  2  2  2  1  1  1  1  1 |

^a^Head Coaches; ^b^Medical personnel; ^c^Players

| **Theme** | **Sub-theme** | **Lower order theme** | **Number of participants** | **Number of quotes** |
| --- | --- | --- | --- | --- |
| Prevention of injuries |  |  |  |  |
|  | Injury prevention strategies |  | 30 (7C^a^; 6M^b^; 17P^c^) | 154 |
|  |  | Mini-band exercises  Training session modification  Recovery (nutrition and sleep)  Jumping and landing  Sprinting and high speed running  Stretching  Proprioception  Mobility exercises  Lower extremity strength exercises  FIFA 11+  Massage  Romanian deadlift  Taping  Eccentric exercises  Valgus knee angle  Nordic curls  Isometric exercises  Copenhagen adductor exercise  Hamstrings quadriceps ratio | 17 (5C; 2M; 10P)  13 (3C; 3M; 7P)  11 (2C; 3M; 6P)  9 (2C; 3M; 4P)  6 (2M; 4P)  6 (2C; 1M; 3P)  5 (2M; 3P)  5 (1C; 2M; 2P)  4 (1M; 3P)  3 (2C; 1M)  3 (1C; 2M)  3 (1M; 2P)  2 (2P)  2 (1C; 1M)  2 (1C; 1M)  1 (1M)  1 (1M)  1 (1M)  1 (1M) | 24  21  23  16  17  8  5  6  5  6  7  4  2  3  2  1  1  1  2 |

^a^Head Coaches; ^b^Medical personnel; ^c^Players

| **Theme** | **Sub-theme** | **Lower order theme** | **Number of participants** | **Number of quotes** |
| --- | --- | --- | --- | --- |
| Injury management |  |  |  |  |
|  | Medical personnel qualifications |  | 10 (2C^a^; 1M^b^; 7P^c^) | 20 |
|  |  | Student medical personnel  Chartered Physiotherapist | 8 (2C; 1M; 5P)  2 (2P) | 17  3 |

^a^Head Coaches; ^b^Medical personnel; ^c^Players

| **Theme** | **Sub-theme** | **Lower order theme** | **Number of participants** | **Number of quotes** |
| --- | --- | --- | --- | --- |
| Injury management |  |  |  |  |
|  | Medical personnel availability |  | 19 (3C^a^; 7M^b^; 9P^c^) | 45 |
|  |  | Availability at training sessions  Medical staff at matches  Availability to assess injury on same day  Remote consultations  Student medical personnel  One medical person  Risk to players due to absence of medical personnel  Showing players that the club cares  Pitch side training availability  Lack of time with patients  Doctor availability | 11 (1C; 4M; 6P)  5 (1M;4P)  4 (1C; 2M; 1P)  3 (3P)  2 (1M; 1M)  2 (1C; 1P)  2 (2P)  1 (1C)  1 (1M)  1 (1M)  1 (1P) | 16  8  5  3  2  3  2  2  1  2  1 |

^a^Head Coaches; ^b^Medical personnel; ^c^Players

| **Theme** | **Sub-theme** | **Lower order theme** | **Number of participants** | **Number of quotes** |
| --- | --- | --- | --- | --- |
| Injury management |  |  |  |  |
|  | Knowledge & competencies of medical personnel |  | 22 (4C^a^; 5M^b^; 13P^c^) | 56 |
|  |  | Return to play strategies  Interpersonal skills  Common injuries  Duty of care to the players  Inexperienced medical personnel  Concussion management  Exercise therapy  Injury prevention strategy knowledge  Evidence-based practice  Open and honest  Training load management  Emergency trauma management | 11 (2M; 9P)  9 (1C; 3M; 5P)  5 (5P)  4 (1C; 1M; 2P)  3 (3P)  2 (1M; 1P)  2 (2P)  2 (2P)  1 (1C)  1 (1C)  1 (1M)  1 (1M) | 12  12  5  6  6  2  3  4  2  2  1  1 |

^a^Head Coaches; ^b^Medical personnel; ^c^Players

| **Theme** | **Sub-theme** | **Lower order theme** | **Number of participants** | **Number of quotes** |
| --- | --- | --- | --- | --- |
| Injury management |  |  |  |  |
|  | Communication between player, coach & medical personnel |  | 26 (6C^a^; 8M^b^; 12P^c^) | 87 |
|  |  | Final communication with player regarding fitness to play  Communication between coach and medical personnel  Communication between coach and medical personnel regarding player availability  Communication between coach and player  Communication between medical personnel and players  Importance of transparency  Ad hoc communication between coach and medical personnel  Trust from coach in effort levels of medical personnel  Coach encouraging autonomy of players  Communication between female medical personnel and players  Communication between male medical personnel and players  Ideal world verus real world communication  Importance of word choice by medical personnel  Medical personnel communication contact availability with players  Trust from players that communicating with medical personnel won’t affect selection | 11 (2M; 9P)  9 (1C; 4M; 5P)  6 (5P)  5 (1C; 1M; 2P)  4 (1M;3P)  4 (1M; 1P)  2 (2P)  1 (1P)  1 (1C)  1 (1C)  1 (1M)  1 (1M)  1 (1M)  1 (1M)  1 (1P) | 23  17  10  11  7  5  2  1  2  1  1  1  3  2  2 |

^a^Head Coaches; ^b^Medical personnel; ^c^Players
